# Circ6834 suppresses non-small cell lung cancer progression by destabilizing ANHAK and regulating miR-873-5p/TXNIP axis

**DOI:** 10.1186/s12943-024-02038-3

**Published:** 2024-06-18

**Authors:** Maoye Wang, Xiaoge Ding, Xinjian Fang, Jing Xu, Yanke Chen, Yu Qian, Jiahui Zhang, Dan Yu, Xiaoxin Zhang, Xiuqin Ma, Taofeng Zhu, Jianmei Gu, Xu Zhang

**Affiliations:** 1 Department of Laboratory Medicine, School of Medicine, Jiangsu University, Zhenjiang, 212013 China; 2 Departmemt of Clinical Laboratory Medicine, Nantong Tumor Hospital/Affiliated Tumor Hospital of Nantong University, Nantong, 226300 China; 3 Department of Pulmonary and Critical Care Medicine, Yixing Hospital affiliated to Jiangsu University, Yixing, 214200 China; 4 Department of Oncology, Gaochun Hospital Affiliated to Jiangsu University, Nanjing, 211300 China

**Keywords:** CircRNA, circ6834, TGF-β, NSCLC, Progression, Therapeutic target

## Abstract

**Background:**

Circular RNAs (circRNAs) play important roles in cancer progression and metastasis. However, the expression profiles and biological roles of circRNAs in non-small cell lung cancer (NSCLC) remain unclear.

**Methods:**

In this study, we identified a novel circRNA, hsa_circ_0006834 (termed circ6834), in NSCLC by RNA-seq and investigated the biological role of circ6834 in NSCLC progression in vitro and in vivo. Finally, the molecular mechanism of circ6834 was revealed by tagged RNA affinity purification (TRAP), western blot, RNA immunoprecipitation, dual luciferase reporter gene assays and rescue experiments.

**Results:**

Our results showed that circ6834 was downregulated in NSCLC tumor tissues and cell lines. Circ6834 overexpression inhibited NSCLC cell growth and metastasis both in vitro and in vivo, while circ6834 knockdown had the opposite effect. We found that TGF-β treatment decreased circ6834 expression, which was associated with the QKI reduction in NSCLC cells and circ6834 antagonized TGF-β-induced EMT and metastasis in NSCLC cells. Mechanistically, circ6834 bound to AHNAK protein, a key regulator of TGF-β/Smad signaling, and inhibited its stability by enhancing TRIM25-mediated ubiquitination and degradation. In addition, circ6834 acted as a miRNA sponge for miR-873-5p and upregulated TXNIP gene expression, which together inactivated the TGF-β/Smad signaling pathway in NSCLC cells.

**Conclusion:**

In conclusion, circ6834 is a tumor-suppressive circRNA that inhibits NSCLC progression by forming a negative regulatory feedback loop with the TGF-β/Smad signaling pathway and represents a novel therapeutic target for NSCLC.

**Supplementary Information:**

The online version contains supplementary material available at 10.1186/s12943-024-02038-3.

## Introduction

Lung cancer is the most common cancer worldwide with an average five-year survival rate of 15–20% [1, 2]. Non-small cell lung cancer (NSCLC) accounts for approximately 85% of all lung cancer cases and can be categorized as adenocarcinoma, squamous cell carcinoma, and large cell carcinoma [3]. Although the diagnosis and treatment of NSCLC have improved, the mortality rate is still high. Thus, it is important to better understand the pathogenesis of NSCLC and identify novel biomarkers and therapeutic targets for NSCLC.

Circular RNAs (circRNAs) are a class of noncoding RNAs characterized by closed-loop structures (4). CircRNAs regulate gene expression by acting as miRNA sponges, regulators of transcription and splicing, protein scaffolds and translation [5–8]. CircRNAs have been reported to be critically involved in the pathological processes of many diseases including cancer. Accumulating studies suggest that circRNAs play important roles in NSCLC development and progression. For instance, circHMGB2 was upregulated in NSCLC and promoted NSCLC cell proliferation and anti-PD-1 resistance via interacting with miR-181a-5p to regulate the expression of CARM1 [9]. Chen et al. demonstrated that circSCAP acted as a tumor suppressor in NSCLC by regulating the ubiquitination of SF3A3 protein and activating p53 signaling [10]. These findings indicate that circRNAs are important regulators of NSCLC and may be potential diagnostic biomarkers and therapeutic targets for NSCLC.

Transforming growth factor β (TGF-β) induces epithelial-mesenchymal transition (EMT) and promotes cancer metastasis by activating the Smad pathway [11]. TGF-β binds to its receptors (TβRI and TβRII) to form a heteromeric complex of serine-threonine kinase receptors to activate Smad2/3. Activated Smad2/3 recruits Smad4, and this protein complex enters the nucleus to regulate EMT-related gene expression [12, 13]. Recently, several studies have suggested that noncoding RNAs regulate TGF-β signaling in EMT. For instance, lncRNA LITATS1 regulated TGF-β-induced EMT by interacting with SMURF2, a key E3 ubiquitin ligase, promoting its cytoplasmic retention and the ubiquitination and proteasomal degradation of TβR1 [14]. CircPTK2 served as a miR-429/miR-200b3p sponge to regulate TIF1γ expression and inhibit TGF-β-induced EMT and tumor metastasis in NSCLC [15]. Circ-DOCK5, whose expression was downregulated by ZEB1, acting as a ‘reservoir’ to increase miR-627-3p stability, which inhibited TGF-β expression and suppressed metastasis in esophageal squamous cell carcinoma [16]. Circ-OXCT1 suppressed gastric cancer EMT and metastasis by attenuating TGF-β pathway through the regulation of miR-136/SMAD4 axis [17]. Intriguingly, a recent study showed that TGF-β signaling promotes cervical cancer metastasis via upregulation of circRNA CDR1as, which facilitated IGF2BP1 binding to Slug mRNA and enhanced its stability [18]. These studies suggest that noncoding RNAs, including circRNAs, may orchestrate a comprehensive regulatory network involving TGF-β signaling to regulate EMT and cancer metastasis.

In this study, we identified a downregulated circRNA circ6834 in NSCLC and demonstrated its inhibitory effect on NSCLC progression both in vitro and in vivo. Interestingly, we found that circ6834 was closely linked to the cellular response to TGF-β stimulation in NSCLC cells. The expression of circ6834 exerted a negative regulatory effect on the TGF-β-induced activation of Smad signaling pathway and subsequent EMT in NSCLC cells, while TGF-β in turn decreased circ6834 expression in NSCLC cells. We demonstrated that circ6834 bound to AHNAK, a positive regulator of TGF-β/Smad signaling pathway and EMT, to enhance its ubiquitination and degradation by TRIM25. Circ6834 also served as a miRNA sponge for miR-873-5p to upregulate the expression of thioredoxin-interacting protein (TXNIP), an inhibitor of TGF-β signaling and EMT. Therefore, circ6834 might act as a potential diagnostic biomarker and therapeutic target for NSCLC.

## Materials and methods

### Patients and tissue samples

Tissue samples were collected from NSCLC patients who underwent surgery at Nantong Tumor Hospital from June 2015 to August 2017. The tissue samples were snap-frozen in liquid nitrogen and stored at -80℃ before RNA extraction. This study was approved by the ethics committee of Jiangsu University. Written informed consent was obtained from all patients. All procedures were carried out in accordance with relevant regulations and guidelines.

### Cell culture

Human NSCLC cell lines (A549, H1299, and PC9), bronchial epithelial cells (HBE) and HEK293T cells were purchased from the Cell Bank of the Chinese Academy of Sciences (Shanghai, China). A549 cells were cultured in DMEM/F12 medium (Meilunbio, Dalian, China). H1299, PC9 and HBE cells were maintained in RPMI 1640 (Meilunbio) and HEK293T cells were kept in DMEM (Meilunbio). 10% fetal bovine serum (ExCell Bio, Suzhou, China) and 1% penicillin-streptomycin (NCM Biotech, Suzhou, China) were supplemented in cell medium. All the cells were incubated in an atmosphere of 5% CO_2_ at 37℃. Cells were starved in media without FBS overnight before the stimulation with recombinant human TGF-β1 (Pepro Tech, USA).

### RNA isolation, reverse transcription and quantitative real-time polymerase chain reaction (qRT-PCR)

Total RNA from tissues and cultured cells was isolated with TRIzol reagent (Invitrogen). Total RNA was reverse-transcribed to cDNA by HiScript III 1st Strand cDNA Synthesis Kit (Vazyme, Nanjing, China). QRT-PCR was performed with SYBR Green Master mix (Vazyme, Nanjing, China) on a Real-time PCR Detection System (Bio-Rad, USA). β-actin was used as the internal control gene. The data were analyzed using the -ΔCt or 2^−ΔΔCt^ method. The sequences of the primers used are listed in Supplementary Table S1.

### Cell transfection

SiRNAs and miRNA mimics were synthesized by GenePharma (Shanghai, China). The circ6834 overexpression plasmid was synthesized by BersinBio Company (Guangzhou, China). NSCLC cells were cultured in 6-well plates (2 × 10^5^/well) overnight and transfected with Lipofectamine 2000 (Invitrogen, Carlsbad, CA, USA) following the manufacturer’s instructions. The medium was changed after 4–6 h, and the cells were cultured for another 24–48 h. The sequences of the siRNAs and miRNA mimics used are listed in Supplementary Table S2.

### Cell counting and colony formation assay

For the cell counting assay, the transfected cells were seeded in 24-well plates (1 × 10^4^/well) and counted every day for 5 days. For the cell colony formation assay, the transfected cells were cultured in 6-well plates (1 × 10^3^/well) for 10 days. The medium was changed every 3 days. The colonies were fixed in 4% paraformaldehyde for 30 min and stained with 0.5% crystal violet for 15 min. The number of colonies was calculated under a microscope (Olympus, Tokyo, Japan). Each experiment was repeated in triplicate.

### Cell migration and invasion assays

Cell migration and invasion assays were carried out using Transwell chambers with 8 μm pore (Corning, NY, USA). The transfected cells were resuspended in 200 µL of serum-free medium and added to the upper chamber. The bottom chamber was filled with 600 µL of complete medium. For cell invasion assay, the upper chamber was precoated with 50 µL of diluted Matrigel (BD Biosciences, New Jersey, USA). After 24–48 h, the cells on the bottom surface were fixed in 4% paraformaldehyde for 30 min and stained with 0.5% crystal violet for 15 min. The number of migrated cells and invaded cells in three randomly selected fields was counted. These experiments were repeated three times.

### Cell cycle and apoptosis analysis

Cell cycle analysis was performed with a cell cycle detection kit (Fcmacs, Jiangsu, China). The transfected cells were fixed in 95% ethanol and then treated with RNase A and propidium iodide (PI). Flow cytometry (BD Biosciences, San Jose, CA, USA) was used to determine the percentage of cells in different phases. For cell apoptosis analysis, the cell apoptosis rate was determined by flow cytometry using an Annexin V-Alexa Fluor 647/PI apoptosis detection kit (Fcmacs, Jiangsu, China).

### Western blot

Cells were lysed using the protein extraction reagent RIPA (Thermo Fisher Scientific, Waltham, MA, USA) with protease and phosphatase inhibitor (Thermo Fisher Scientific). The extracted proteins were separated by 10% SDS-polyacrylamide gel and transferred to 0.45 μm PVDF membranes (Millipore, Billerica, MA, USA). After blocking with 5% non-fat milk, the membranes were probed with primary antibodies against E-Cadherin, N-Cadherin, vimentin, Snail, Slug, Bcl-2, Cyclin D1, Smad2/3, p-Smad2, p-Smad3 (Cell Signaling Technology, Shanghai, Danvers, MA, USA), GAPDH (Abclonal, Wuhan, China), TRIM25, TXNIP (Proteintech, Wuhan, China), and AHNAK (Santa Cruz Biotechnology, Santa Cruz, CA) overnight at 4℃, followed by incubation with their corresponding secondary antibody (Thermo Fisher Scientific). A chemiluminescence system (GE, USA) was used to visualize the target proteins.

### Animal models

BALB/c nude mice (4 weeks old, male) were obtained from Cavens (Changzhou, China). Circ6834 overexpression and control cells (1 × 10^6^) were resuspended in 200 µL of PBS and subcutaneously injected into mice (5 mice/group). Tumor size was examined every 3 days during the animal experiment. Tumor volumes (V) were calculated using the formula: V = 1∕2×length×width^2^ (mm^3^). For tumor lung metastasis, the transfected cells (5 × 10^5^) suspended in 100 µL of PBS were injected into the tail vein of nude mice. At 4–6 weeks post-injection, the mice were sacrificed and the tumors and lungs were removed and photographed. Consecutive sections were made and stained with hematoxylin and eosin (H&E). The protocol was approved by the Animal Use and Care Committee of Jiangsu University.

### Immunofluorescence and immunohistochemistry

Tumors were cut into 4-µm sections and incubated with primary monoclonal antibodies against Ki-67, N-cadherin, E-cadherin and Vimentin followed by incubation with the secondary antibody at room temperature according to the instructions (Boster, Wuhan, China). Then, DAB staining and counterstaining with hematoxylin were carried out (Boster, Wuhan, China). Finally, images of the sections were obtained with a Pannoramic MIDI (3D HISTECH, Budapest, Hungary).

### Tagged RNA affinity purification assay

A tagged RNA affinity purification (TRAP) assay was performed as previously described [19]. In brief, NSCLC cells were cotransfected with control and circ6834 overexpression vectors containing MS2 stem-loop structure (MS2 and circRNA-MS2) and GST-MS2 overexpression vector (Bersinbio, China). The proteins potentially interacting with circ6834 were identified with a TRAP Kit (Bersinbio) and subjected to mass spectrometry analysis. The results of the TRAP experiment were further verified by western blot.

### Co-immunoprecipitation assay (Co-IP)

Cell lysates were incubated with antibodies and magnetic beads (Thermo, Waltham, MA) at 4 ℃ overnight and washed with cell lysis buffer. The co-immunoprecipitated proteins were eluted from the magnetic beads for western blot analysis.

### RNA sequencing

Total RNA was extracted from paired NSCLC tissues and adjacent nontumor tissues, or from control and circ6834-overexpressing NSCLC cells and sequenced by OEBiotech (Shanghai, China). Differentially expressed genes (DEGs) were screened and identified mainly through the set |log2 (FoldChange)| and *P* values.

### RNA immunoprecipitation assay

RNA immunoprecipitation (RIP) was performed to validate the interaction of circ6834 with Ago2 via a PureBinding-RNA Immunoprecipitation kit (GENESEED, Guangzhou, China). NSCLC cells were lysed in RIP lysis buffer with magnetic beads and 5 µg of the antibody of interest (human anti-Ago2 antibody and negative control IgG). After RNA extraction, reverse transcription and qRT-PCR were used to detect and analyze the results.

### Dual-luciferase reporter assay

The luciferase reporter vectors containing wild type (WT) or mutant (MUT) of circ6834 and miR-873-5p mimics were cotransfected into HEK293T cells or NSCLC cells using Lipofectamine 2000 (Invitrogen, Carlsbad, CA, USA) on. At 24 h after transfection, cells were lysed and collected, and the luciferase activities were determined by the Dual Luciferase Assay System (Promega, Madison, WI, USA).

### Statistical analysis

All the data were presented as mean ± standard deviation (SD). SPSS 22.0 (Chicago, IL, USA) or GraphPad Prism 7.0 (La Jolla, CA, USA) software was used to analyze the data. Student’s *t* test and ANOVA were used to elevate the differences between different groups. Pearson’s χ^2^ test was used for the clinical variables. Survival analysis was performed using the Kaplan-Meier method and evaluated with log-rank test. The area under the ROC curve (AUC) was analyzed to estimate the effectiveness of circ6834 for prediction. *P* < 0.05 was considered statistically significant.

## Results

### Identification and validation of dysregulated circRNAs in NSCLC

**Fig. 1:**
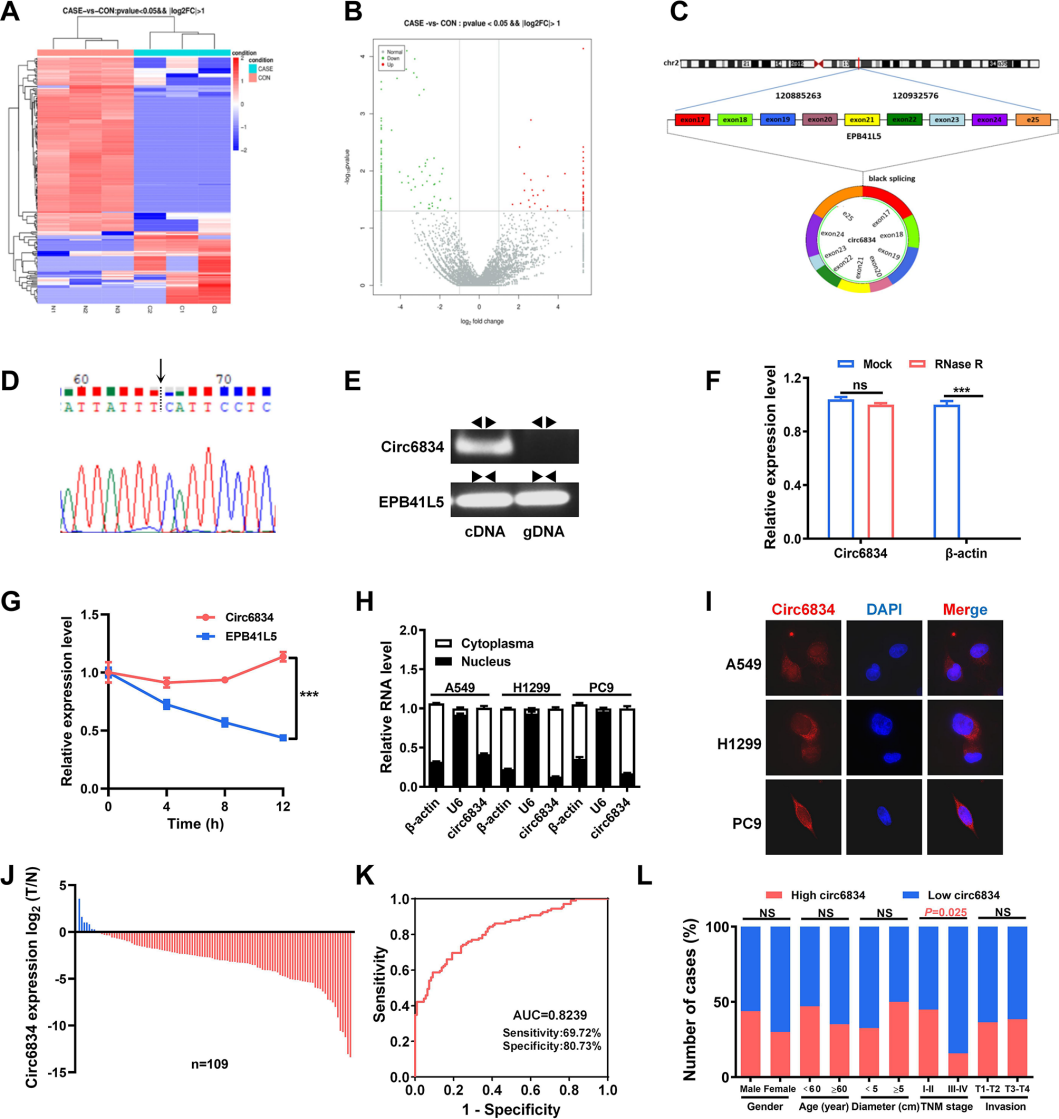
Circ6834 is downregulated in NSCLC. **A-B** Heatmap (**A**) and volcano plot (**B**) of differentially expressed circRNAs in NSCLC tissues and adjacent normal ones. **C** Schematic illustration of circ6834 biosynthesis. **D** Sanger sequencing validation of the backsplice junction site of circ6834 in the PCR product. **E** PCR and agarose gel electrophoresis analysis of the presence of circ6834 and EPB41L5 by divergent and convergent primers. **F** Expression of circ6834 and β-actin in NSCLC cells with or without RNase R treatment. **G** Analysis for alterations in circ6834 and EPB41L5 after treatment with actinomycin D. **H-I** Detection of the subcellular localization of circ6834 in NSCLC cells by RNA nuclear/cytosol fractionation (**H**) and FISH (**I**). **J** QRT-PCR was performed to examine the expression of circ6834 in paired NSCLC and non-tumor tissues (*n* = 109). **K** ROC curve of circ6834 in distinguishing NSCLC and normal tissues. **L** Graphical illustration of the statistical circ6834 distribution in NSCLC patients

RNA sequencing was conducted using tumor tissues and adjacent non-tumor tissues from three NSCLC patients to assess differences in the circRNA expression profile in NSCLC. The hierarchical clustering and volcano plot revealed 187 differentially expressed circRNAs (55 upregulated and 132 downregulated) between NSCLC tissues and noncancerous tissues (Fig. 1A, B). According to GO and KEGG analyses, the differentially expressed circRNAs involved in tumor cell proliferation and metastasis were listed and verified by qRT-PCR (Supplementary Fig. 1A, B). We focused on circ6834 because this circRNA has not been previously reported in human NSCLC. Circ6834 is transcribed from the host gene EPB41L5 by back-splicing of exons 17 to 25 with 958 nucleotides, which was validated by Sanger sequencing of the PCR product (Fig. 1C, D). Moreover, the results of PCR with divergent primers showed that circ6834 was amplified from cDNA but not from the genomic DNA of NSCLC cells (Fig. 1E). The results of actinomycin D and RNase R exonuclease treatment showed that circ6834 was resistant to their digestion (Fig. 1F, G). Collectively, these data supported that circ6834 was a circular RNA with high stability.

To assess the subcellular distribution of circ6834 in NSCLC cells, we conducted RNA nuclear/cytosol fractionation and fluorescence in situ hybridization (FISH) experiments. Both results showed that circ6834 was mainly localized in the cytoplasm of NSCLC cells (Fig. 1H, I). Furthermore, qRT-PCR was performed to detect the expression of circ6834 in the tissues of NSCLC patients and NSCLC cell lines. We found that circ6834 expression was downregulated in NSCLC tissues and cell lines (A549, H1299, and PC9) compared to paired noncancerous tissues and bronchial epithelial cells (HBE), respectively (Fig. 1J and Supplementary Fig. 1A). The receiver operating characteristic (ROC) curve was used to investigate the diagnostic value of circ6834 in NSCLC with AUC of 0.8239 (Fig. 1K). We then evaluated the clinical significance of circ6834 in NSCLC and found that low levels of circ6834 were related to advanced TNM stage in NSCLC patients (Fig. 1L). In addition, NSCLC patients with low circ6834 expression seem to have a shorter survival time than those who had higher expression (Supplementary Fig. 1C). These data indicate that the downregulation of circ6834 may be involved in NSCLC progression.

### Circ6834 overexpression inhibits NSCLC growth and metastasis

**Fig. 2:**
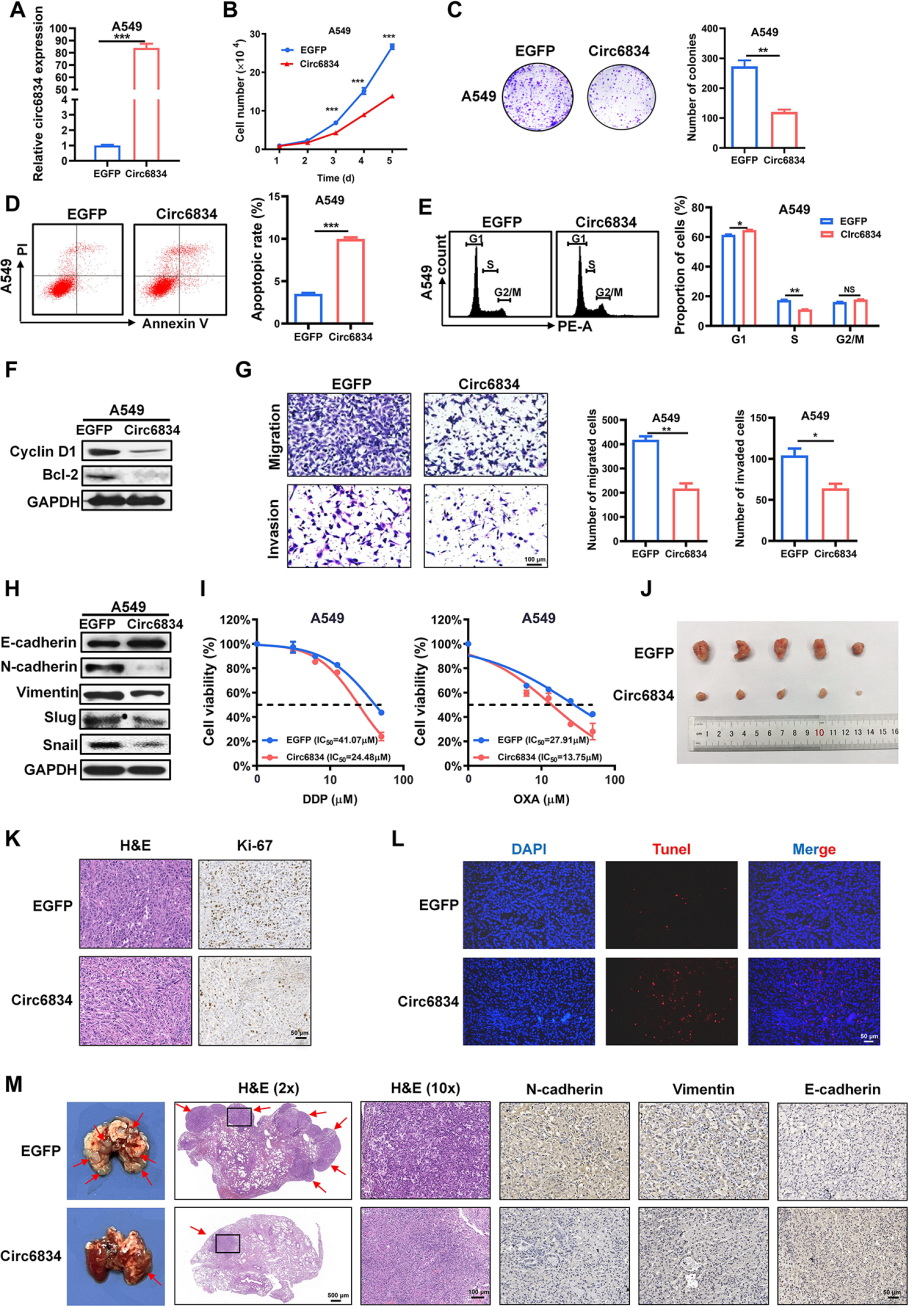
Circ6834 overexpression suppresses NSCLC growth and metastasis in vitro and in vivo. **A** The efficiency of circ6834 overexpression in A549 cells. **B-E, G** Cell growth curves (**B**), colony formation assays (**C**), flow cytometric analyses of cell apoptosis (**D**) and cell cycle (**E**), and Transwell migration and Matrigel invasion assays (**G**) for EGFP and circ6834-overexpressing A549 cells. **F, H** Western blot analyses for the expression of Cyclin D1, Bcl-2 (**F**), and EMT markers (**H**) in EGFP and circ6834-overexpressing A549 cells. **I** The effects of circ6834 overexpression on the sensitivity of A549 cells to DDP and OXA treatment. **J** Images of tumors from mice in the EGFP and circ6834-overexpressing groups. **K-L** H&E, Ki-67 (**K**) and TUNEL (**L**) staining of mouse tumor tissues from the EGFP and circ6834-overexpressing groups. **M** Representative images and IHC staining of lung tissues with metastatic modules from mice in the EGFP and circ6834-overexpressing groups

We next performed gain-of- and loss-of-function studies to determine the biological roles of circ6834 in NSCLC progression. The efficiency of circ6834 overexpression was verified by qRT-PCR (Fig. 2A and Supplementary Fig. 2A). The results of cell growth curves and colony formation assays showed that circ6834 overexpression inhibited NSCLC cell proliferation (Fig. 2B, C and Supplementary Fig. 2B, C). We also observed that NSCLC cells with circ6834 overexpression exhibited increased apoptosis and decreased S phase (Fig. 2D, E and Supplementary Fig. 2D, E). Subsequently, the protein levels of cyclin D1 and Bcl-2 were significantly decreased in NSCLC cells with circ6834 overexpression (Fig. 2F and Supplementary Fig. 2F). The results of Transwell assays showed that circ6834 overexpression inhibited NSCLC cell migration and invasion (Fig. 2G and Supplementary Fig. 2G). Moreover, circ6834 overexpression promoted the acquisition of epithelial phenotypes in NSCLC cells. Circ6834 overexpression significantly upregulated E-cadherin expression, and downregulated the expression of N-cadherin, Vimentin, Slug and Snail in NSCLC cells (Fig. 2H and Supplementary Fig. 2H). ​In addition, we evaluated the effect of circ6834 overexpression on the sensitivity of NSCLC cells to chemotherapy, such as cisplatin (DDP) and Oxaliplatin (OXA). As expected, circ6834 overexpression remarkably enhanced the sensitivity of NSCLC cells to DDP or OXA treatment (Fig. 2I and Supplementary Fig. 2I). To further verify the effects of circ6834 on the malignant progression of NSCLC, we knocked down circ6834 expression in NSCLC cells by using siRNAs that target the back-splice junction site of circ6834 (Supplementary Fig. 3A). Compared with those in the si-NC group, the proliferation, migration, and invasion abilities of NSCLC cells were markedly enhanced in the si-circ6834 group (Supplementary Fig. 3B-E).

We then analyzed the effect of circ6834 on NSCLC growth and metastasis in vivo. The results of the mouse xenograft tumor model showed that circ6834 overexpression significantly inhibited both tumor size and tumor weight (Fig. 2J and Supplementary Fig. 4A-C). The decreased number of proliferative tumor cells and increased number of apoptotic cells in the circ6834-overexpressing group were confirmed by Ki-67 and TUNEL staining assays (Fig. 2K, L). To explore the role of circ6834 in tumor metastasis in vivo, we established a lung metastasis model by tail-vein injection. Compared to the control group, circ6834 overexpression significantly suppressed lung metastasis in NSCLC cells. H&E staining further indicated a reduction of metastatic modules in mouse lung tissues from circ6834-overexpressing group. Moreover, immunohistochemical analysis demonstrated decreased expression of EMT markers, such as N-cadherin and Vimentin, while E-cadherin expression was increased in mouse lung tissues from circ6834-overexpressing group (Fig. 2M). Collectively, these results suggested that circ6834 effectively suppressed growth and metastasis of NSCLC both in vitro and in vivo.

### Circ6834 responds to TGF-β stimulus and suppresses TGF-β-induced EMT

**Fig. 3:**
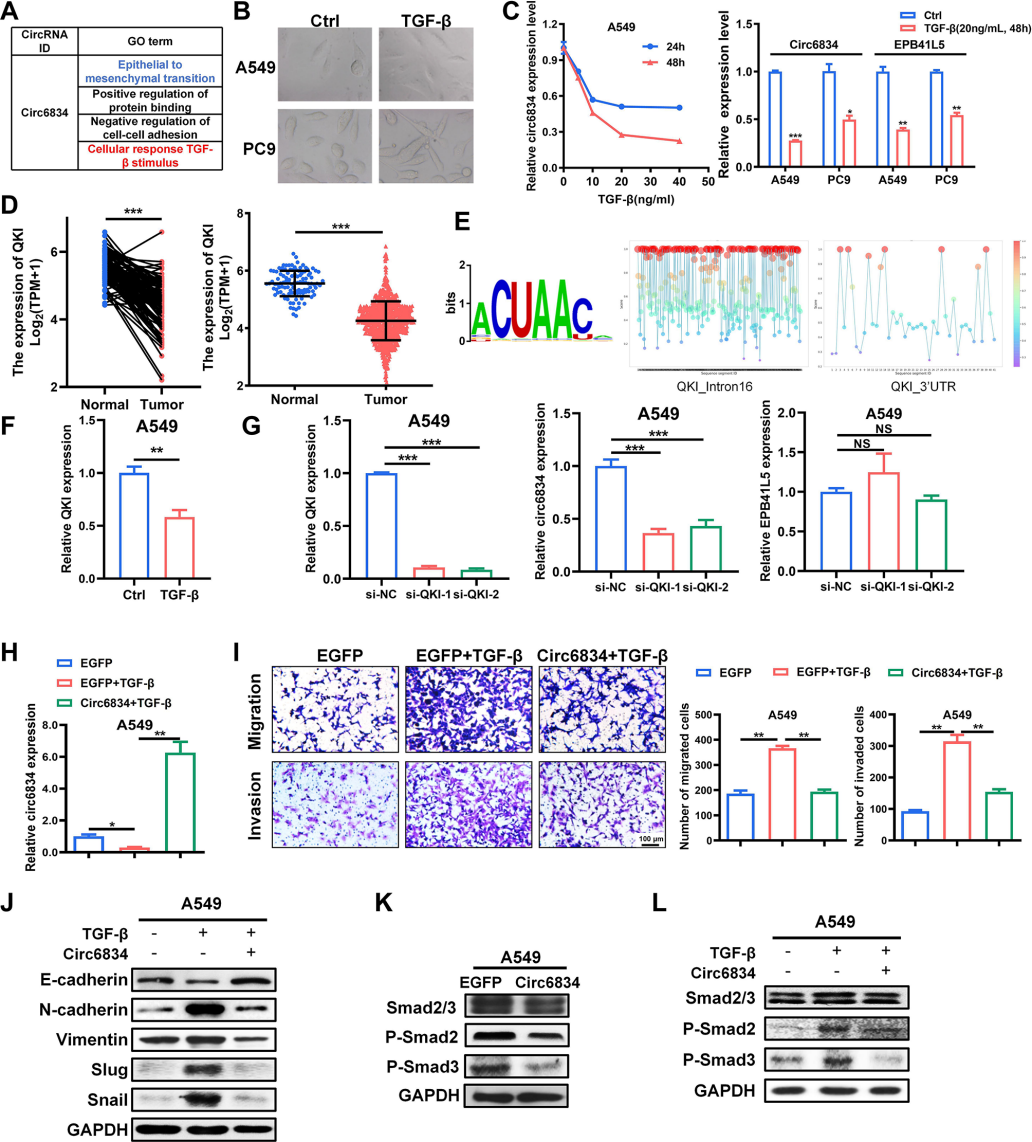
Circ6834 responds to TGF-β stimulus and inhibits TGF-β/Smad pathway in NSCLC cells. **A** GO analysis of circ6834. **B** Representative images of NSCLC cell morphology before and after TGF-β treatment. **C** Relative circ6834 and EPB41L5 expression in NSCLC cells after TGF-β treatment. **D** TCGA analysis of QKI expression in lung cancer tissues and normal ones. **E** A schematic view of QKI binding motif (left) and potential QRE motifs in EPB41L5 pre-mRNA predicted by RBPsuite (right). **F** Relative QKI expression in A549 cells with TGF-β treatment detected by qRT-PCR. **G** QRT-PCR for QKI, circ6834, and EPB41L5 expression in QKI knockdown A549 cells. **H-I** QRT-PCR for circ6834 (**H**) and Transwell migration and Matrigel invasion assays (**I**) in A549 cells overexpressing circ6834 and treated with TGF-β. **J-L** Western blot for EMT markers (**J**) and p-Smad2/3 expression (**K, L**) in A549 cells with different treatment

GO analysis suggested that circ6834 is involved in the cellular response to TGF-β and associated with TGF-β/Smad signaling pathway and EMT in NSCLC cells (Fig. 3A). To verify this, we first treated NSCLC cells with TGF-β1. The morphology of NSCLC cells changed to a mesenchymal phenotype after TGF-β1 treatment (Fig. 3B). The qRT-PCR results showed that circ6834 expression was markedly decreased in NSCLC cells treated with TGF-β1 in a time- and dose-dependent manner (Fig. 3C). In addition, EPB41L5 level also decreased when treated with TGF-β1 (Fig. 3C).

It has been widely reported that RNA-binding protein quaking (QKI) can regulate circRNA biogenesis by binding to intronic QKI response elements (QREs) during the EMT process [20]. Thus, we attempted to explore whether the regulation of circ6834 expression by TGF-β was dependent on QKI. We found that QKI was significantly downregulated in NSCLC tissues (Fig. 3D). QRE was defined as a bipartite consensus sequence NACUAAY-N_1 − 20_-UAAY, containing a core sequence NACUAAY (Y, pyrimidine) and a neighboring half-site UAAY separated by 1–20 nt [21]. Many potential QRE motifs were found in the intron flanking the circ6834-forming exons and 3’UTR after the last exon by RBPsuite website [22] (Fig. 3E). After treatment with TGF-β, both circ6834 and QKI expression decreased (Fig. 3C, F and Supplementary Fig. 5A). We next inhibited QKI expression via siRNAs and found that circ6834 rather than EPB41L5 expression dropped accompanied by QKI decrease (Fig. 3G and Supplementary Fig. 5B). These results indicated that QKI was associated with the reduction of circ6834 expression by TGF-β treatment.

It is widely known that TGF-β induces cancer metastasis via EMT while circ6834 was confirmed to inhibit NSCLC metastasis. Therefore, we wanted to explore whether circ6834 could reverse the effects of TGF-β1 treatment (Fig. 3H and Supplementary Fig. 5C). As shown in Fig. 3I and Supplementary Fig. 5D, circ6834 overexpression abolished TGF-β1-induced migration and invasion of NSCLC cells. Moreover, we found that TGF-β1 treatment decreased the protein level of E-cadherin and increased those of N-cadherin, Vimentin, Slug and Snail, while these effects were reversed by circ6834 overexpression (Fig. 3J and Supplementary Fig. 5E). Smad proteins are pivotal intracellular effectors of TGF-β1 [23]. Thus, we detected the status of Smad2 and Smad3 in NSCLC cells overexpressing circ6834 and treated with TGF-β1. The phosphorylation of both Smad2 and Smad3 decreased in circ6834-overexpressing NSCLC cells but increased in those with circ6834 knockdown (Fig. 3K and Supplementary Fig. 3G, 5 F). Upon TGF-β1 treatment, the levels of p-Smad2 and p-Smad3 increased in NSCLC cells while this trend was reversed by circ6834 overexpression (Fig. 3L and Supplementary Fig. 5G). Taken together, these findings suggest that circ6834 is a regulator of TGF-β-induced EMT and metastasis in NSCLC.

### Circ6834 interacts with AHNAK protein

**Fig. 4:**
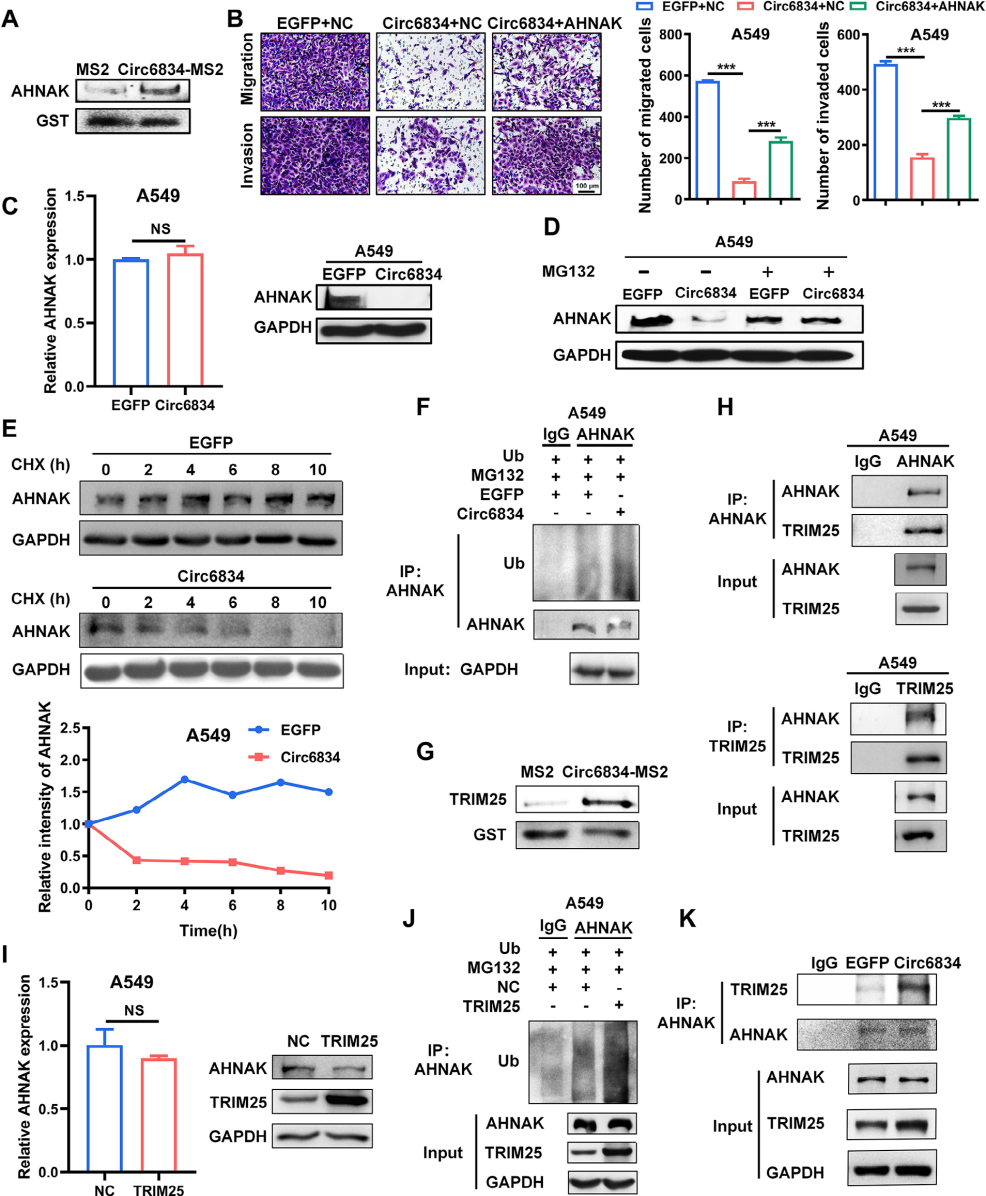
Circ6834 promotes AHNAK ubiquitination via TRIM25. **A** Verification of the interaction between circ6834 and AHNAK by TRAP and western blot. **B** Transwell migration and Matrigel invasion assays of A549 cells cotransfected with circ6834 and AHNAK overexpression plasmids. **C** AHNAK mRNA and protein levels in A549 cells overexpressing circ6834. **D** MG132 (40 µM, 6 h) treatment for AHNAK expression in A549 cells overexpressing circ6834. **E** CHX assay for determining the half-life of AHNAK protein in A549 cells overexpressing circ6834. **F** The levels of ubiquitinated AHNAK in A549 cells overexpressing circ6834. **G** The association between AHNAK and TRIM25 was verified by TRAP and western blot. **H** Co-IP assay for determining the binding between AHNAK and TRIM25. **I** AHNAK mRNA and protein levels in TRIM25-overexpressing A549 cells. **J** The level of ubiquitinated AHNAK in A549 cells overexpressing TRIM25. **K** The binding ability of TRIM25 to AHNAK was determined by co-IP assay after circ6834 overexpression

To further investigate the mechanism of circ6834 in NSCLC, we carried out a tagged RNA affinity purification (TRAP) assay to detect target proteins binding to circ6834. The potential binding proteins were analyzed by LC-MS/MS (Supplementary Fig. 6A-D). We chose AHNAK as the candidate protein, which has been reported as an important regulator of TGF-β-induced EMT [24, 25]. Knockdown of AHNAK inhibited the metastatic ability of NSCLC cells (Supplementary Fig. 6E-H). We verified the interaction between circ6834 and AHNAK protein by another independent TRAP assay combined with western blot (Fig. 4A). More importantly, AHNAK overexpression reversed circ6834 overexpression-mediated inhibition of NSCLC cell migration and invasion (Fig. 4B and Supplementary Fig. 7A).

In addition, we found that AHNAK protein levels were significantly decreased while AHNAK mRNA levels exhibited minimal changes in NSCLC cells after circ6834 overexpression (Fig. 4C and Supplementary Fig. 7B). Similarly, AHNAK protein level also decreased in circ6834 overexpression group of lung metastasis model (Supplementary Fig. 7C). Protein stability experiments showed that proteasome inhibitor MG132 abolished the reduction in AHNAK protein levels induced by circ6834 overexpression (Fig. 4D and Supplementary Fig. 7D). We transfected NSCLC cells with circ6834 plasmid, treated them with cycloheximide (CHX) and then monitored the half-life of AHNAK protein. The results showed that circ6834 overexpression obviously promoted AHNAK protein degradation in NSCLC cells (Fig. 4E and Supplementary Fig. 7E).

To verify whether circ6834 could potentiate AHNAK degradation via a ubiquitination-dependent pathway, we cotransfected NSCLC cells with circ6834 overexpression plasmids and ubiquitin plasmids, and then treated them with MG132. Compared to EGFP control, circ6834 overexpression led to increased levels of ubiquitinated AHNAK (Fig. 4F). To further analyze the mechanism by which circ6834 promotes AHNAK ubiquitination, we screened the LC-MS/MS results and chosen TRIM25, an E3 ubiquitin ligase, and identified their interaction by TRAP assay. The results of the independent TRAP assay and western blot showed that TRIM25 could be detected among proteins coprecipitated with circ6834 (Fig. 4G). In addition, co-IP assays showed that AHNAK could bind to TRIM25 (Fig. 4H). Furthermore, we confirmed that TRIM25 overexpression decreased AHNAK protein but not mRNA levels, and promoted AHNAK ubiquitination (Fig. 4I, J). More importantly, circ6834 could act as a scaffold to enhance the binding ability between AHNAK and TRIM25 (Fig. 4K). Collectively, these data suggest that circ6834 may interact with AHNAK and promote its ubiquitination-mediated degradation by TRIM25 in NSCLC cells.

### Circ6834 acts as a miR-873-5p sponge

**Fig. 5:**
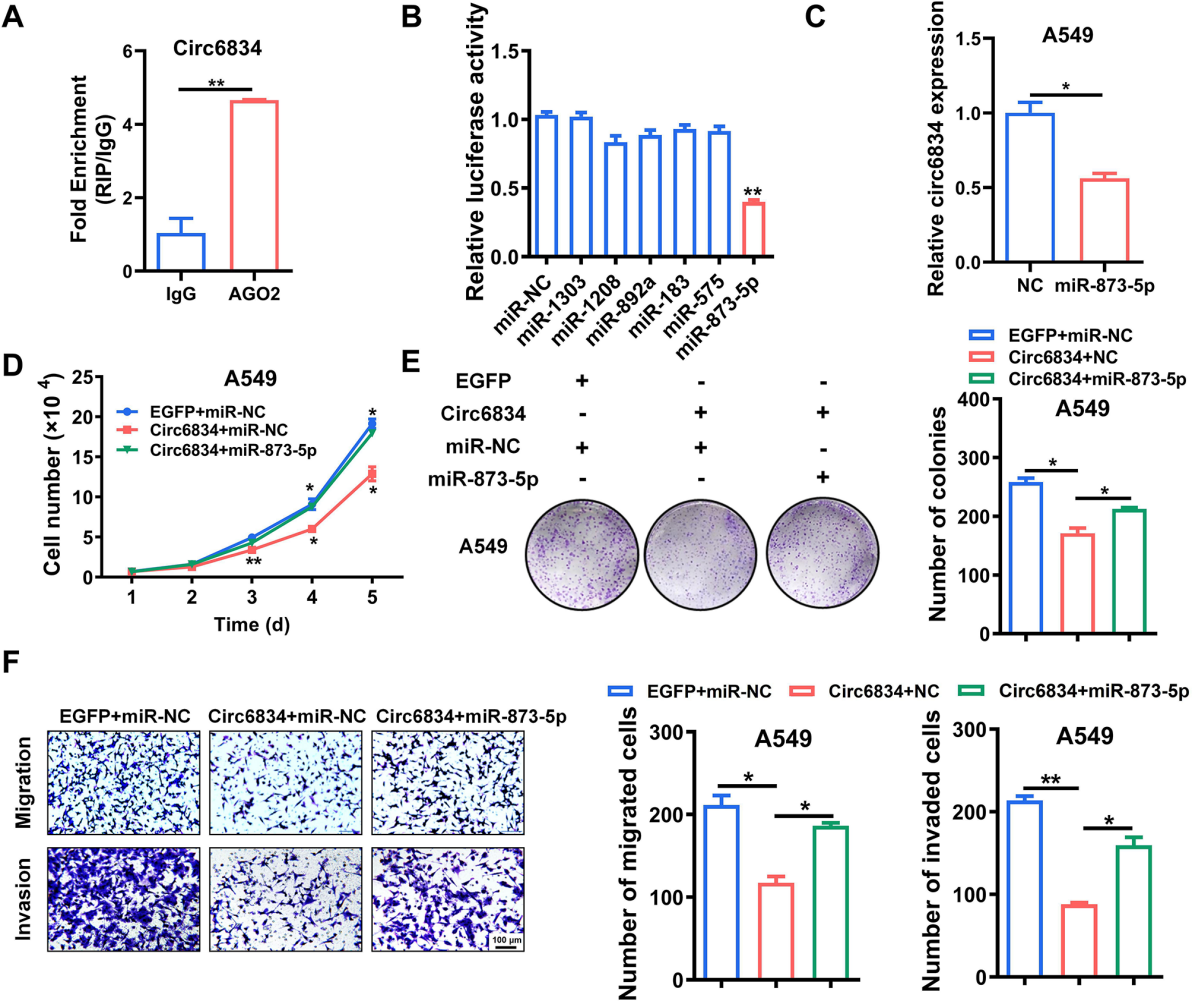
Circ6834 acts as a miRNA sponge for miR-873-5p. **A** RIP assay for the interaction of circ6834 with AGO2 protein. **B** Luciferase reporter assays were performed to screen for potential circ6834-interacting miRNAs. **C** QRT-PCR analysis of circ6834 expression in A549 cells transfected with miR-873-5p mimics. **D-F** Cell growth curves (**D**), colony formation assays (**E**), and Transwell migration and Matrigel invasion assays (**F**) for A549 cells in different groups

CircRNAs have been demonstrated to act as miRNA sponges to regulate gene expression in cancer. RIP assays confirmed that circ6834 could interact with AGO2 protein in NSCLC cells (Fig. 5A). We then predicted the possible circ6834-interacting miRNAs by using the bioinformatic database miRWalk. Based on the predicted results, several miRNAs were chosen and verified via dual-luciferase reporter assays. The results showed that miR-873-5p was one of the candidate miRNAs that most significantly decreased the activity of wild-type circ6834 luciferase reporter (Fig. 5B). The results of qRT-PCR also suggested that miR-873-5p overexpression decreased the expression of circ6834 in NSCLC cells (Fig. 5C).

We further determined the biological roles of miR-873-5p in NSCLC progression and found that miR-873-5p mimics promoted NSCLC cell proliferation, migration, and invasion (Supplementary Fig. 8A-C). To explore the importance of miR-873-5p in circ6834-regulated tumor suppression in NSCLC, miR-873-5p mimics were cotransfected with circ6834 overexpression plasmid into NSCLC cells. The proliferation and metastasis abilities of NSCLC cells were enhanced by miR-873-5p mimics compared to those of cells transfected with circ6834 alone (Fig. 5D-F and Supplementary Fig. 8D-F), indicating that miR-873-5p overturned the suppressive effects of circ6834 on NSCLC cells. Moreover, TCGA database revealed that lung cancer patients with higher miR-873-5p levels had shorter disease-specific survival (DSS), suggesting a good predictive value for the prognosis of patients with NSCLC (Supplementary Fig. 8G).

### Circ6834 regulates miR‑873-5p/TXNIP axis

**Fig. 6:**
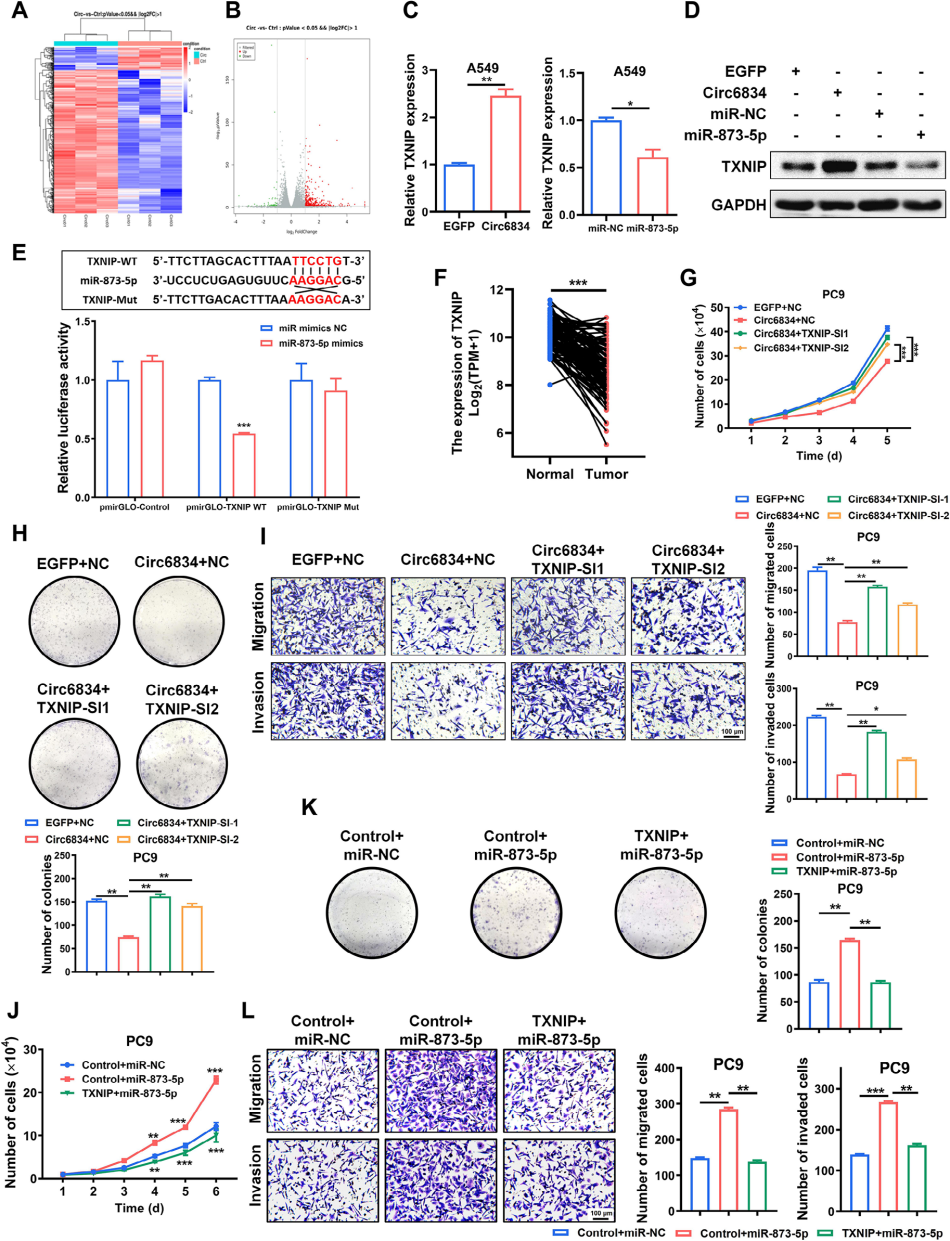
Circ6834 regulates miR-873-5p/TXNIP axis in NSCLC cells. **A-B** Heatmap (**A**) and volcano plot (**B**) of DEGs in the conntrol and circ6834-overexpressing groups. **C-D** QRT-PCR (**C**) and western blot (**D**) were performed to detect the expression of TXNIP in A549 cells with circ6834 or miR-873-5p overexpression. **E** HEK293T cells were transfected with TXNIP WT or Mut plasmids and miR-873-5p mimics as indicated. Relative luciferase activity was detected and normalized to that of the control. **F** TCGA data analysis of TXNIP expression in lung cancer tissues and paired normal ones. **G-I** Cell growth curves (**G**), colony formation assays (**H**), and Transwell migration and Matrigel invasion assays (**I**) of PC9 cells cotransfected with circ6834 plasmid and TXNIP siRNAs. **J-L** Cell growth curves (**J**), colony formation assays (**K**), and Transwell migration and Matrigel invasion assays (**L**) of PC9 cells cotransfected with TXNIP plasmid and miR-873-5p mimics

To identify the target genes of miR-873-5p that are regulated by circ6834, we scanned the genes overlapped between our RNA-seq data and miRWalk predictions and selected TXNIP as the downstream target of miR-873-5p (Fig. 6A, B). TXNIP mRNA and protein levels were upregulated in the circ6834-overexpressing group but decreased in the miR-873-5p mimics group compared to those in the control group (Fig. 6C, D and Supplementary Fig. 9A, B). Furthermore, we verified the regulatory effect of miR-873-5p on TXNIP by using luciferase reporter assays. We cotransfected miR-873-5p mimics with TXNIP WT or Mut luciferase reporter vectors into HEK293T and NSCLC cells. We observed that compared with the control, miR-873-5p inhibited the luciferase activity of reporter genes containing TXNIP WT but had no effect on TXNIP Mut (Fig. 6E and Supplementary Fig. 9C). TXNIP has been previously considered as a tumor suppressor gene [26, 27]. Consistently, TCGA data analysis also confirmed that TXNIP expression was downregulated in NSCLC tissues compared to normal tissues (Fig. 6F). Thus, we explored the effect of TXNIP on circ6834/miR-873-5p axis in NSCLC progression. Knockdown of TXNIP reversed the inhibition of NSCLC cell proliferation, migration and invasion by circ6834 overexpression (Fig. 6G-I and Supplementary Fig. 9D-F), while TXNIP overexpression reversed the promotion of these malignant progressions by miR-873-5p mimics (Fig. 6J-L and Supplementary Fig. 9G-I). Taken together, these results indicate that circ6834 inhibits NSCLC progression via regulation of miR-873-5p/TXNIP axis.

## Discussion

Although tremendous progress has been made in the diagnosis and treatment of NSCLC, the prognosis of patients with late-stage disease remains unsatisfactory. Accumulating studies have shown that circRNAs play important roles in the development and progression of various cancers. To further understand the roles of circRNAs in NSCLC progression, we performed a whole transcriptome resequencing by using paired tumor tissues and adjacent normal tissues from NSCLC patients. In this study, we analyzed the circRNA expression profile of NSCLC and identified 187 differentially expressed circRNAs. We found that circ6834 expression was significantly downregulated in NSCLC tissues and associated with TNM stage. During our study in preparation, another study by Lv et al. reported that circ6834 (termed as circ-EPB41L5, also back-spliced by exon 17 to 25 of EPB41L5) was downregulated in glioblastoma tissues and its low level was associated with poor prognosis in glioblastoma patients. They confirmed that circ6834 inhibited glioblastoma progression via circ6834/miR-19a/EPB41L5/p-AKT regulatory axis [28]. Our findings are consistent with this study, indicating that circ6834 downregulation may be linked to the progression of different cancers.

Previous studies have identified various circRNAs that are dysregulated in NSCLC. For instance, circRNA_102231 expression was evidently upregulated in lung adenocarcinoma tissues and related to the advanced TNM stage, lymph node metastasis, and poor overall survival of lung cancer patients [29]. Li et al. reported that circSATB2 promoted the proliferation, migration, and invasion of NSCLC cells by regulating miR-362/FSCN1 axis [30]. Circ_0078767 was downregulated in NSCLC tissues and its overexpression suppressed NSCLC cell viability, cell cycle progression and EMT while promoting cell apoptosis [31]. In addition, circFGFR1 [32], circNDUFB2 [33], circFARSA [34], and other circRNAs have been reported to be involved in NSCLC proliferation and metastasis via distinct mechanisms. Herein, we reported the biological roles of circ6834 in NSCLC both in vitro and in vivo. Mechanistically, circ6834 potentiated AHNAK degradation via TRIM25 and functioned as a regulator of miR-873-5p/TXNIP, together leading to the inactivation of TGF-β/Smad signaling pathway in NSCLC cells. These findings suggested that circ6834 may serve as a tumor suppressor circRNA to inhibit NSCLC progression, which provides a potential target for NSCLC therapy.

TGF-β induces EMT and promotes metastasis in cancers including NSCLC. Since circRNAs are critical regulators of gene expression, it is unsurprising that they are also involved in TGF-β pathway activation and EMT. For example, circRNA cESRP1 bound to miR-93-5p to upregulate the expression of Smad7/21, which in turn inhibited TGF-β-induced EMT [35]. Li et al. reported that TGF-β/Smad pathway could increase the expression of circ-E-Cad to promote gastric cancer cell proliferation, migration, and EMT [36]. Moreover, circRNA expression controlled by TGF-β could form a regulatory feedback loop. For instance, circPTK2 was downregulated during TGF-β-induced EMT in NSCLC cells and its overexpression inhibits TGF-β-induced EMT and metastasis by enhancing TIF1γ expression [15]. Another study by Zheng et al. revealed that overexpression of circPTEN1 dramatically decreased the metastatic activities induced by TGF-β1. Mechanistically, circPTEN1 inhibited TGF-β-mediated metastasis by disrupting TGF-β/Smad signaling through binding the MH2 domain of Smad4 to disrupt its physical interaction with p-Smad2/3. Our data also showed that circ6834 was downregulated by TGF-β treatment and its overexpression repressed TGF-β-induced EMT and metastatic ability of NSCLC cells. Thus, circ6834 may be another circRNA that can respond to TGF-β and regulate TGF-β-induced EMT in NSCLC. Targeting circ6834 or its downstream effectors in the TGF-β/Smad pathway may have therapeutic potential in NSCLC. Considering that TGF-β is often highly expressed in cancers, this may explain the downregulation of circ6834 in NSCLC. The underlying mechanism by which TGF-β downregulates circ6834 expression is not clear. For further exploration, we found that this effect might be associated with the downregulation of QKI in TGF-β-treated NSCLC cells, as QKI has been previously reported to regulate circRNA abundance during TGF-β-mediated EMT in human mammary cells [20]. Another explanation for the downregulation of circ6834 by TGF-β may be linked to the activation of Smad2/3 signaling pathway. Xu et al. reported that lncRNA SMASR (smad3-associated long non-coding RNA) was downregulated by TGF-β via Smad2/3 in lung cancer cells. Smad2/3 proteins could bind to the promoter region of SMASR gene and TGF-β treatment further increased this occupancy [37]. We also predicted the potential transcription factors that could bind to the promoter region of EPB41L5 gene and found that Smad2/3 was in the predicted results. Whether this mechanism is also responsible for the downregulation of circ6834 deserves further investigation.

The interaction between circRNAs and proteins has been implicated as a key mechanism for cancer progression. CircFOXK2 promoted pancreatic ductal adenocarcinoma (PDAC) progression by interacting with YBX1 and hnRNPK proteins to upregulate the expression of NUF2 and PDXK oncogenes [38]. Liang et al. demonstrated that circDCUN1D4 interacted with HuR protein to mediate its translocation to the cytoplasm, where HuR bound to the mRNA of TXNIP to increase its stability, leading to the inhibition of metastasis in lung adenocarcinoma [39]. Herein, our proteomic analysis revealed that circ6834 bound to AHNAK protein. AHNAK is a giant scaffolding protein of approximately 700 kDa [40] and both tumor-promoting and -suppressive roles of AHNAK have been reported, indicating that AHNAK may function in a cancer type- and cell context-specific manner [41–44]. Interestingly, it was reported that AHNAK could stimulate Smad3 localization into nucleus and potentiate TGF-β signaling [45, 46]. In this study, we found that circ6834 could interact with AHNAK and induce TRIM25-mediated ubiquitination and degradation of AHNAK. We confirmed that AHNAK overexpression reversed circ6834-mediated suppression of NSCLC cell migration and invasion. These results suggest that circ6834 inhibits TGF-β-induced EMT and NSCLC progression by targeting AHNAK.

The function of circRNAs as miRNA sponges has been widely studied in cancer. For example, circHIPK3 could bind to 9 miRNAs with 18 binding sites, including tumor suppressor miR-124, to promote cell growth [47]. CircFOXK2 interacted with miR-942 to upregulate the expression of ANK1, GDNF and PAX6 to promote PDAC growth and metastasis [38]. The circRNA-miRNA-mRNA regulatory axis also plays an important role in NSCLC. CircRNA 100146 targeted miR-361-3p and miR-615-5p to enhance NSCLC cell proliferation, migration, and invasion via regulation of multiple downstream mRNAs [48]. CircTP63 acted as a miR-873-3p sponge to increase FOXM1 expression, which in turn promoted lung squamous cell carcinoma progression [49]. In this study, we found that circ6834 contained binding sites for miR-873-5p and verified their interaction in NSCLC cells. Previous studies have shown that miR-873-5p acted as an oncogene or tumor suppressor in different cancer types [50–52]. Luo et al. reported that miR-873-5p expression was dramatically increased in NSCLC tissues [53]. We demonstrated that miR-873-5p overexpression promoted NSCLC cell proliferation, migration, and invasion and that miR-873-5p cotransfection partially rescued the suppressive effect of circ6834 on NSCLC progression, indicating that circ6834 may serve as a ceRNA of miR-873-5p in NSCLC. TXNIP was identified as a tumor suppressor in many cancers and its downregulation was proposed to promote EMT and cancer metastasis [54, 55]. In melanoma, miR-224/452-mediated downregulation of TXNIP is essential for E2F1-induced EMT and invasion [56]. Moreover, Cheng et al. reported that lncRNA LHFPL3-AS2 interacted with SFPQ to upregulate TXNIP expression, leading to the suppression of NSCLC metastasis [57]. Masaki et al. reported that TXNIP deficiency enhanced TGF-β signaling and EMT in NSCLC cells [58]. Consistent with these studies, we found that circ6834 upregulated TXNIP to inhibit NSCLC progression, which further supports the notion that TXNIP is an important regulator of TGF-β signaling and has potent role in restraining tumor metastasis and EMT.

**Fig. 7:**
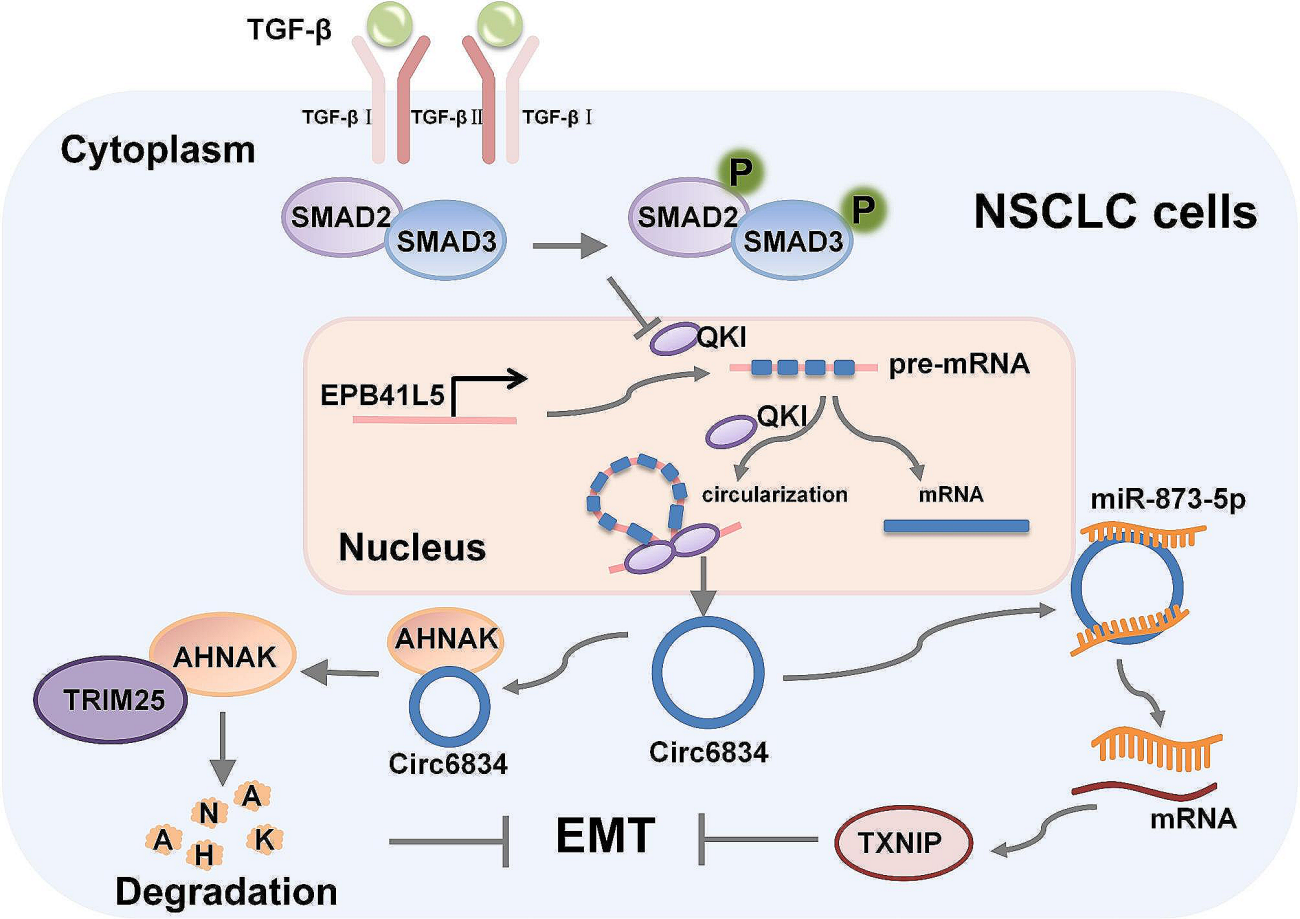
Proposed model for the role of circ6834 in NSCLC progression. Circ6834 inhibits TGF-β-induced Smad pathway activation and EMT via AHNAK/TRIM25 and miR-873-5p/TXNIP axis in NSCLC cells. In addition, TGF-β induces circ6834 downregulation via decreasing QKI

Overall, we found that circ6834 was downregulated in NSCLC and that circ6834 overexpression inhibited NSCLC growth and metastasis. Circ6834 expression was downregulated by TGF-β treatment and QKI knockdown. Meanwhile, circ6834 suppressed TGF-β-induced Smad pathway activation and EMT. Circ6834 exerted these effects by inducing TRIM25-mediated ubiquitination and degradation of AHNAK and acting as a miR-873-5p sponge to upregulate TXINP expression (Fig. 7). These findings reveal that circ6834 functions as a tumor suppressor in NSCLC and represents a potential target for NSCLC therapy.

### Electronic supplementary material

Below is the link to the electronic supplementary material.

Supplementary Material 1

## Data Availability

No datasets were generated or analysed during the current study.
