# Supplementary material for: Circ6834 suppresses non-small cell lung cancer progression by destabilizing ANHAK and regulating miR-873-5p/TXNIP axis
Source: Mol Cancer. 2024 Jun 18;23:128. doi: 10.1186/s12943-024-02038-3 (PMC11184876; doi:10.1186/s12943-024-02038-3)
Supplement: Supplementary file 1 — Supplementary Material 1 [file 12943_2024_2038_MOESM1_ESM.docx]

**Supplementary figures**


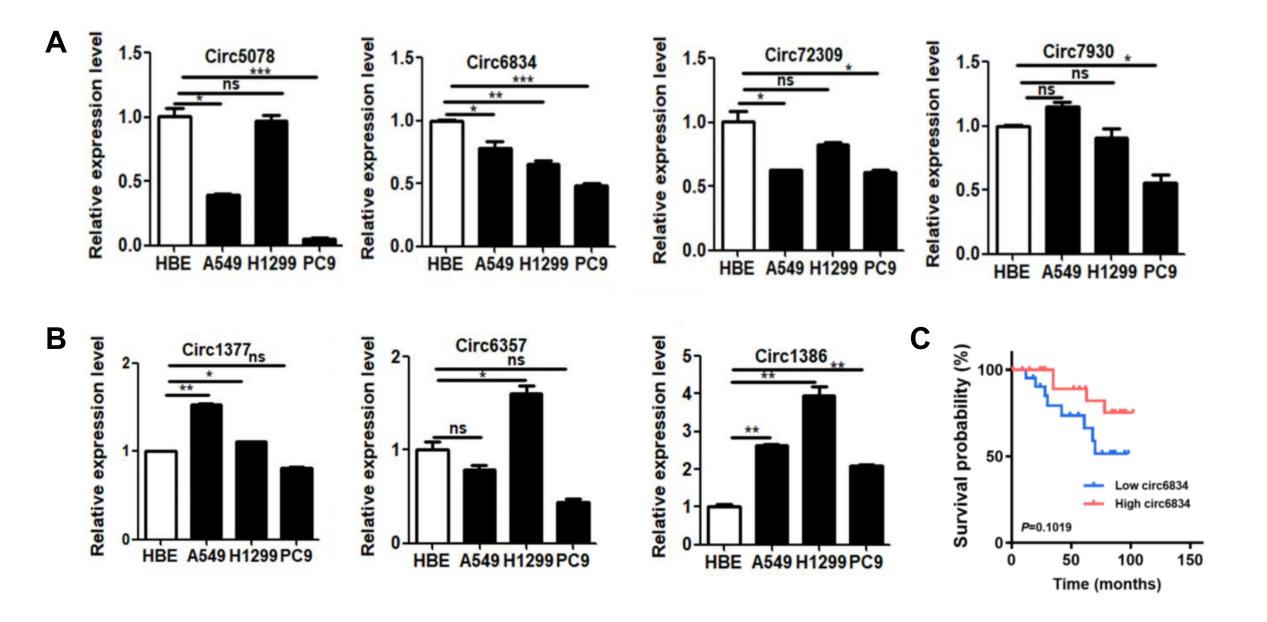


**Supplementary Fig. 1** Expression and prognosis of circRNAs in NSCLC. **A** The expression of downregulated circRNAs (circ5078, circ6834, circ72309, circ7930) in NSCLC cells by qRT-PCR. **B** The expression of upregulated circRNAs (circ1377, circ6357, circ1386) in NSCLC cells by qRT-PCR. **C** Kaplan-Meier analysis of the OS rate in NSCLC patients with high or low circ6834 expression.


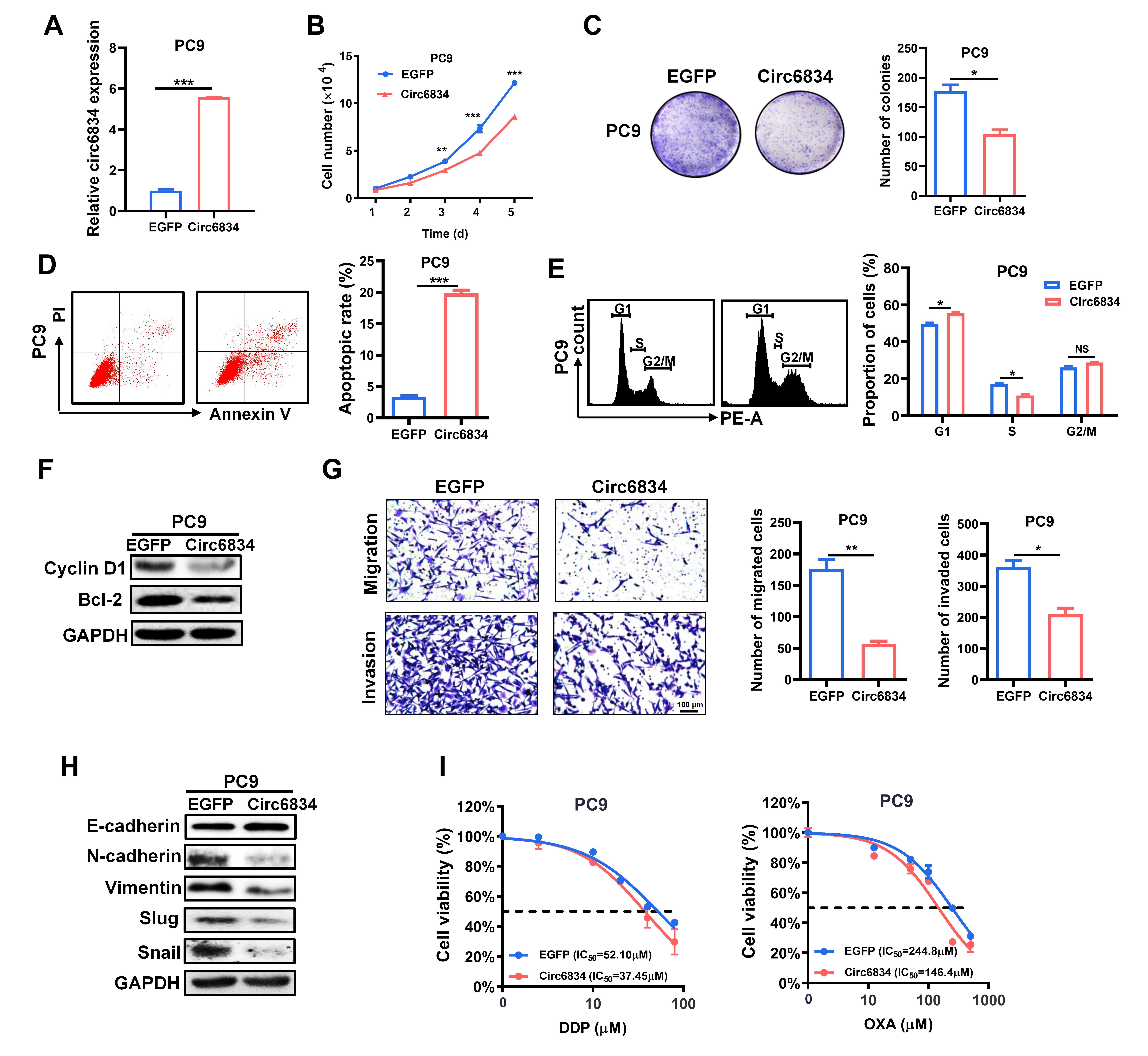


**Supplementary Fig. 2** Circ6834 overexpression inhibits NSCLC malignant biological function. **A** The efficiency of circ6834 overexpression in PC9 cells. **B-E, G** Cell growth curves (**B**), colony formation assays (**C**), flow cytometric analyses of cell apoptosis (**D**) and cell cycle (**E**), and Transwell migration and Matrigel invasion assays (**G**) for EGFP and circ6834-overexpressing PC9 cells. **F, H** Western blot analyses for the expression of Cyclin D1, Bcl-2 (**F**), and EMT markers (**H**) in EGFP and circ6834-overexpressing PC9 cells. **I** The effects of circ6834 overexpression on the sensitivity of PC9 cells to DDP and OXA treatment.


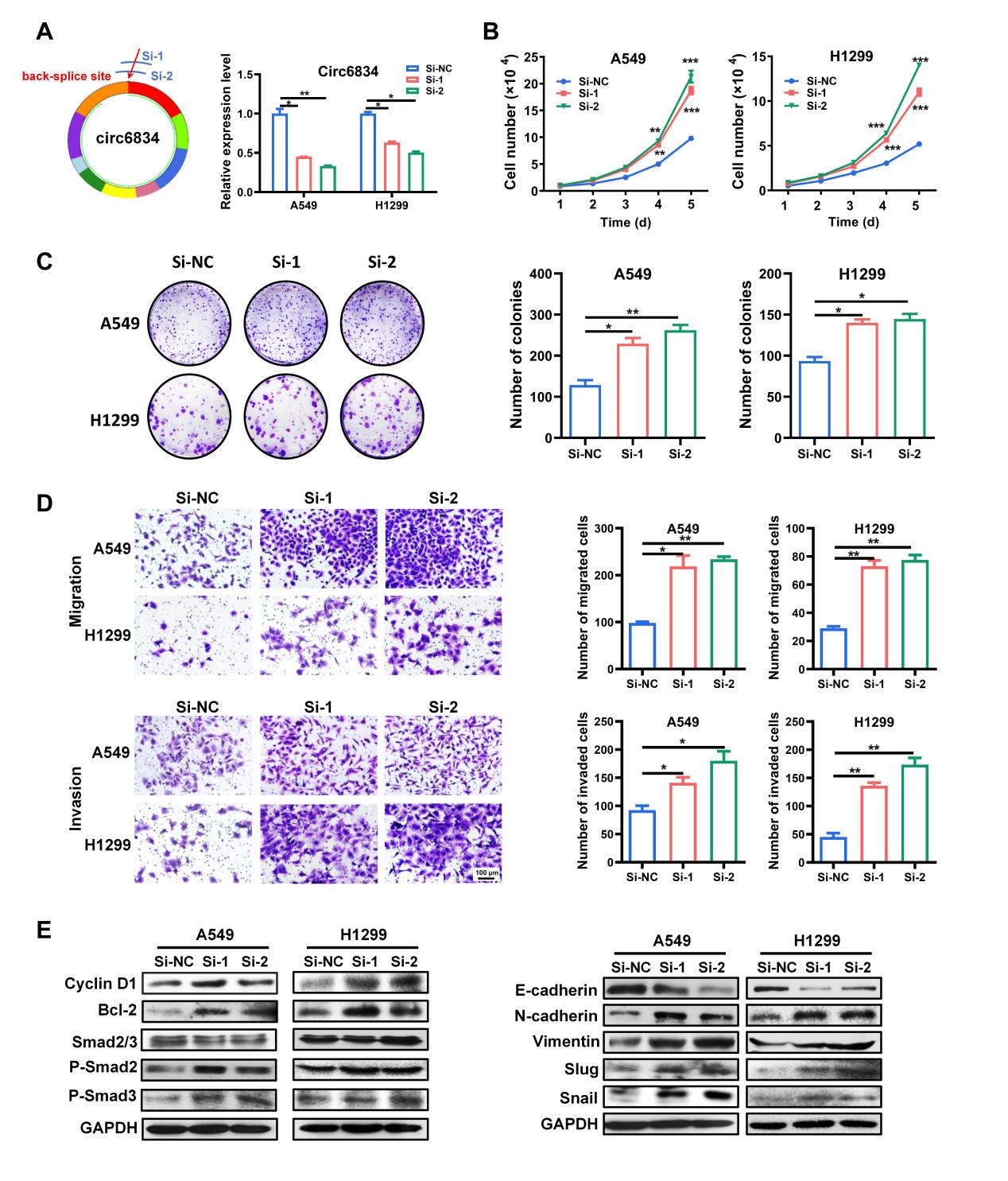


**Supplementary Fig. 3** Circ6834 knockdown promotes NSCLC cell proliferation, migration, and invasion. **A** Schematic diagram and efficiency of circ6834 knockdown via an RNAi technique. **B-D** Cell growth curves (**B**), colony formation (**C**), and Transwell migration and Matrigel invasion assays (**D**) for Si-NC and circ6834 knockdown NSCLC cells. **E** Western blot analyses for the expression of Cyclin D1, Bcl-2, EMT markers and phospho-Smad2/3 in Si-NC and circ6834 knockdown NSCLC cells.


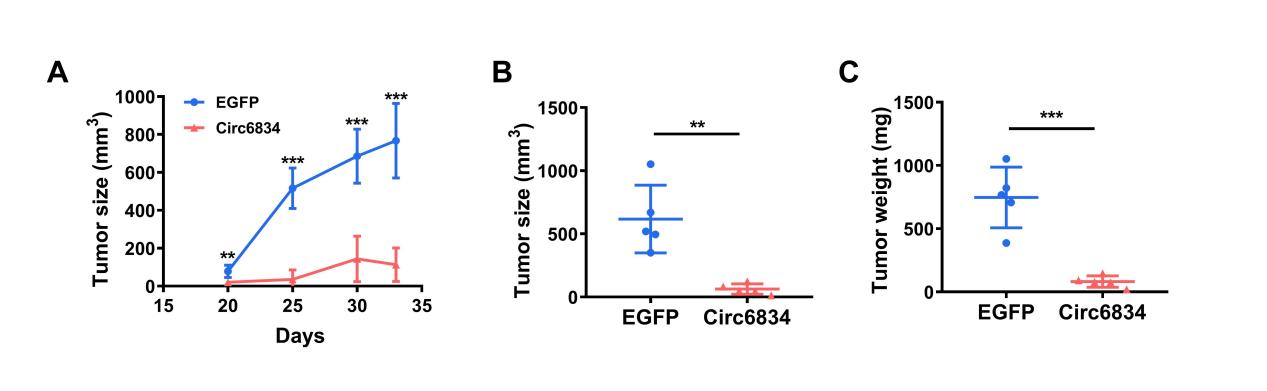


**Supplementary Fig. 4** Circ6834 overexpression suppresses NSCLC growth in vivo. **A-C** Growth curves (A), volumes (B), and weights (C) of tumors from mice in the EGFP and circ6834-overexpressing groups.


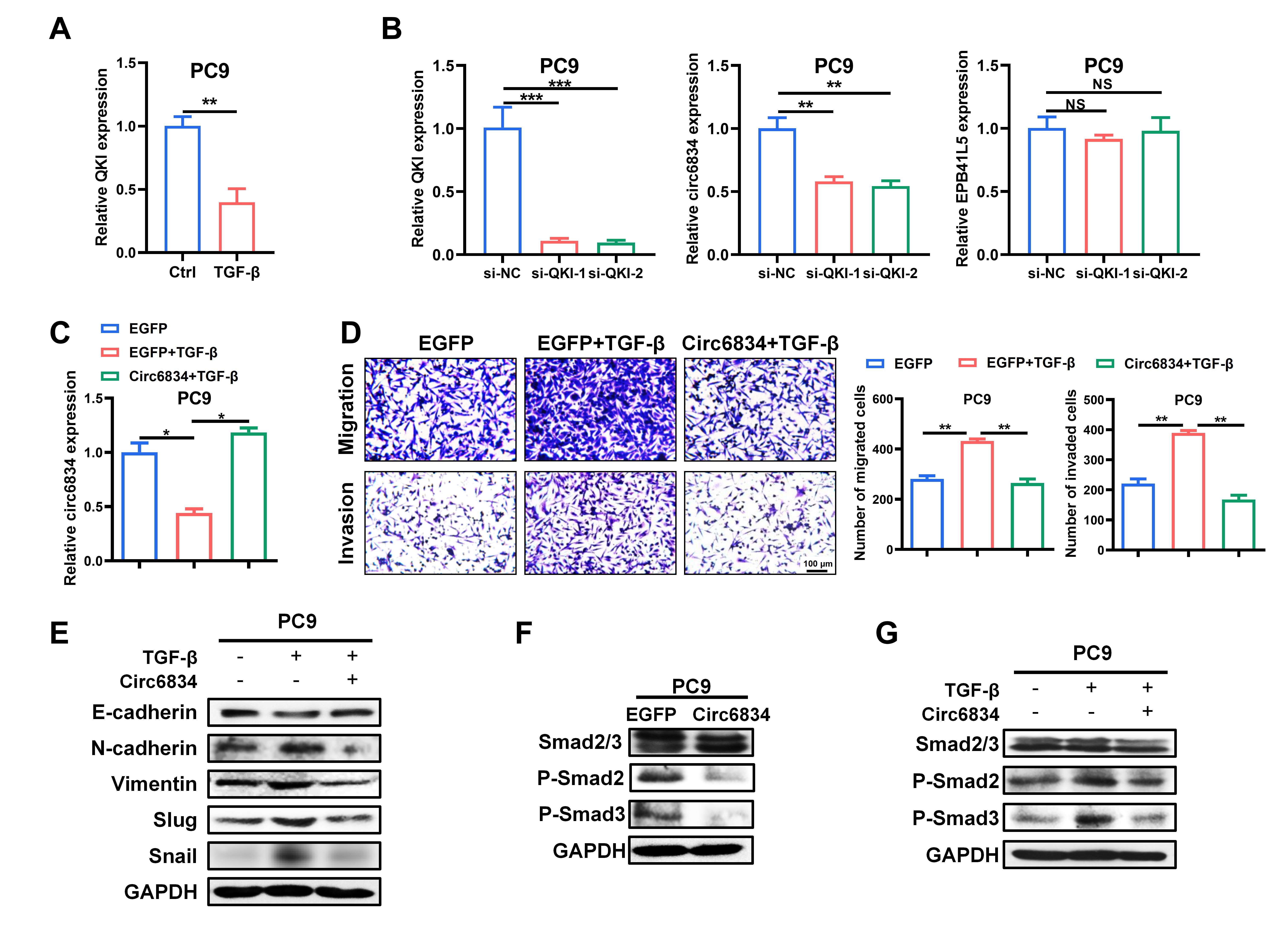


**Supplementary Fig. 5** Circ6834 responds to TGF-β stimulus and inhibits TGF-β/SMAD pathway in PC9 cells. **A** Relative QKI expression in PC9 cells after TGF-β treatment detected by qRT-PCR. **B** QRT-PCR for QKI, circ6834, and EPB41L5 expression in QKI knockdown PC9 cells. **C-D** QRT-PCR for circ6834 (**C**), and Transwell migration and Matrigel invasion assays (**D**) in PC9 cells overexpressing circ6834 treated with TGF-β. **E-G** Western blot for EMT markers (**E**), and p-SMAD2/3 expression (**F, G**) in PC9 cells with different treatments.


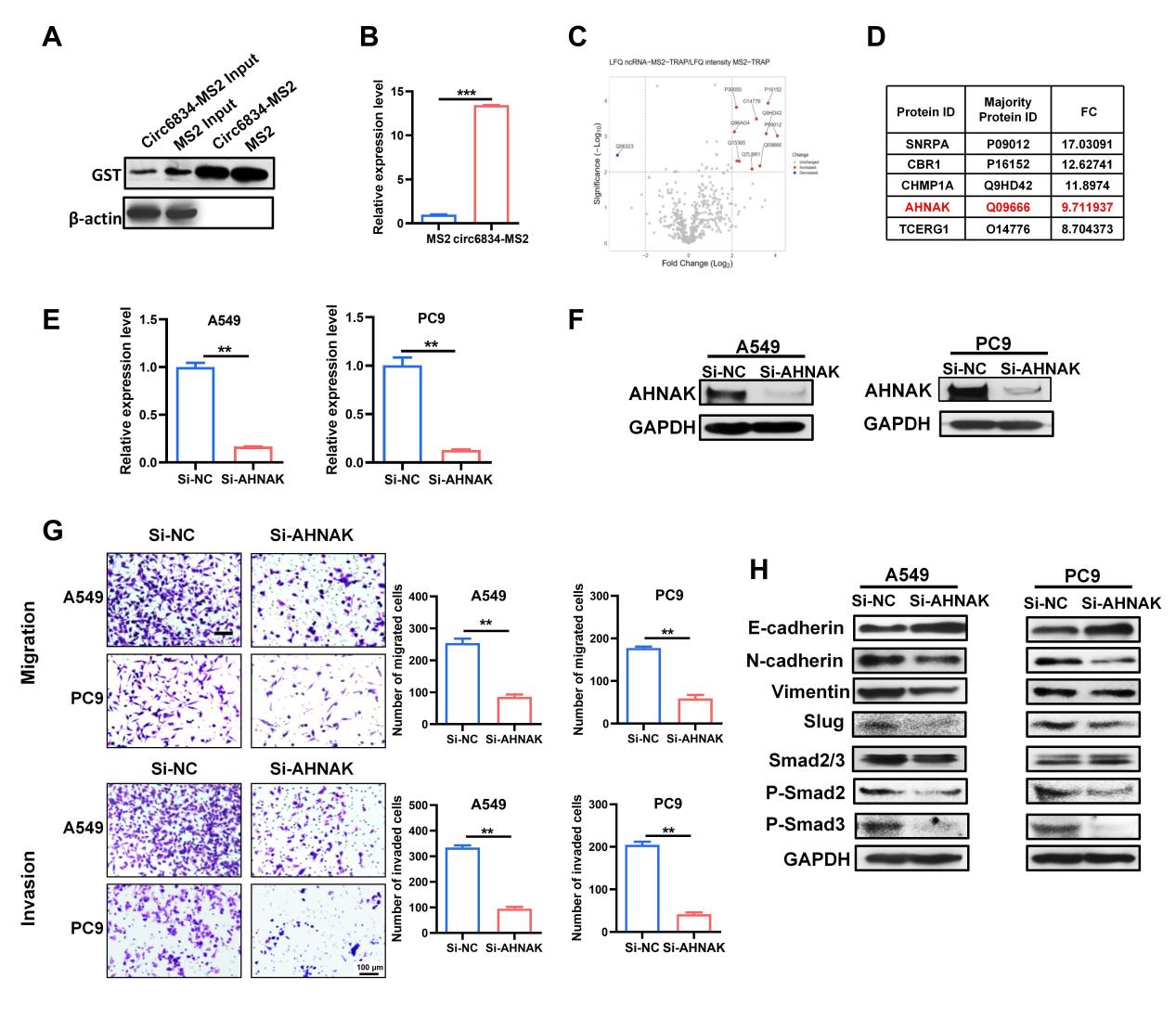


**Supplementary Fig. 6** AHNAK knockdown inhibits NSCLC cell metastasis. **A** GST protein expression was detected by western blot. **B** QRT-PCR was conducted to detect circ6834 expression in MS2 and circ6834-MS2 group. **C-D** The volcano plot (**C**) and list (**D**) of circ6834 binding proteins analyzed by LC-MS/MS. **E-F** The RNA (**E**) and protein (**F**) level of AHNAK in knockown NSCLC cells. **G** Transwell migration and Matrigel invasion assays of AHNAK knockown NSCLC cells. **H** Western blot analyses for the expression of EMT markers and P-Smad2/3 in AHNAK knockown NSCLC cells.


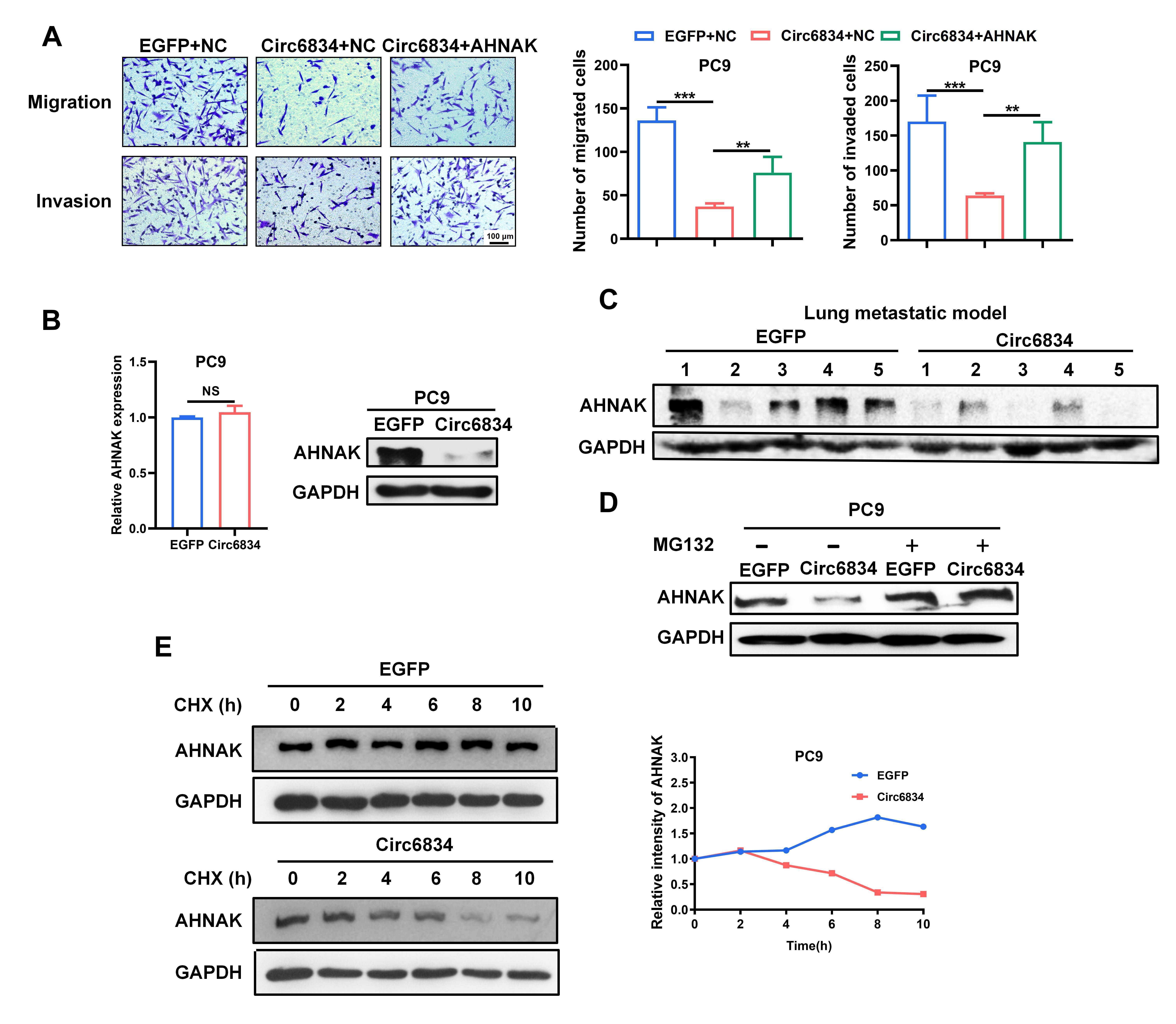


**Supplementary Fig. 7** Circ6834 promotes AHNAK ubiquitination. **A** Transwell migration and Matrigel invasion assays for PC9 cells cotransfected with circ6834 and AHNAK. **B** AHNAK mRNA and protein levels in PC9 cells overexpressing circ6834. **C** Western blot for AHNAK expression in lung metastasis tissues. **D** MG132 (40 μM, 6 h) experiment for AHNAK expression in PC9 cells overexpressing circ6834. **E** CHX assay for determining the half-life of AHNAK protein in PC9 cells overexpressing circ6834.


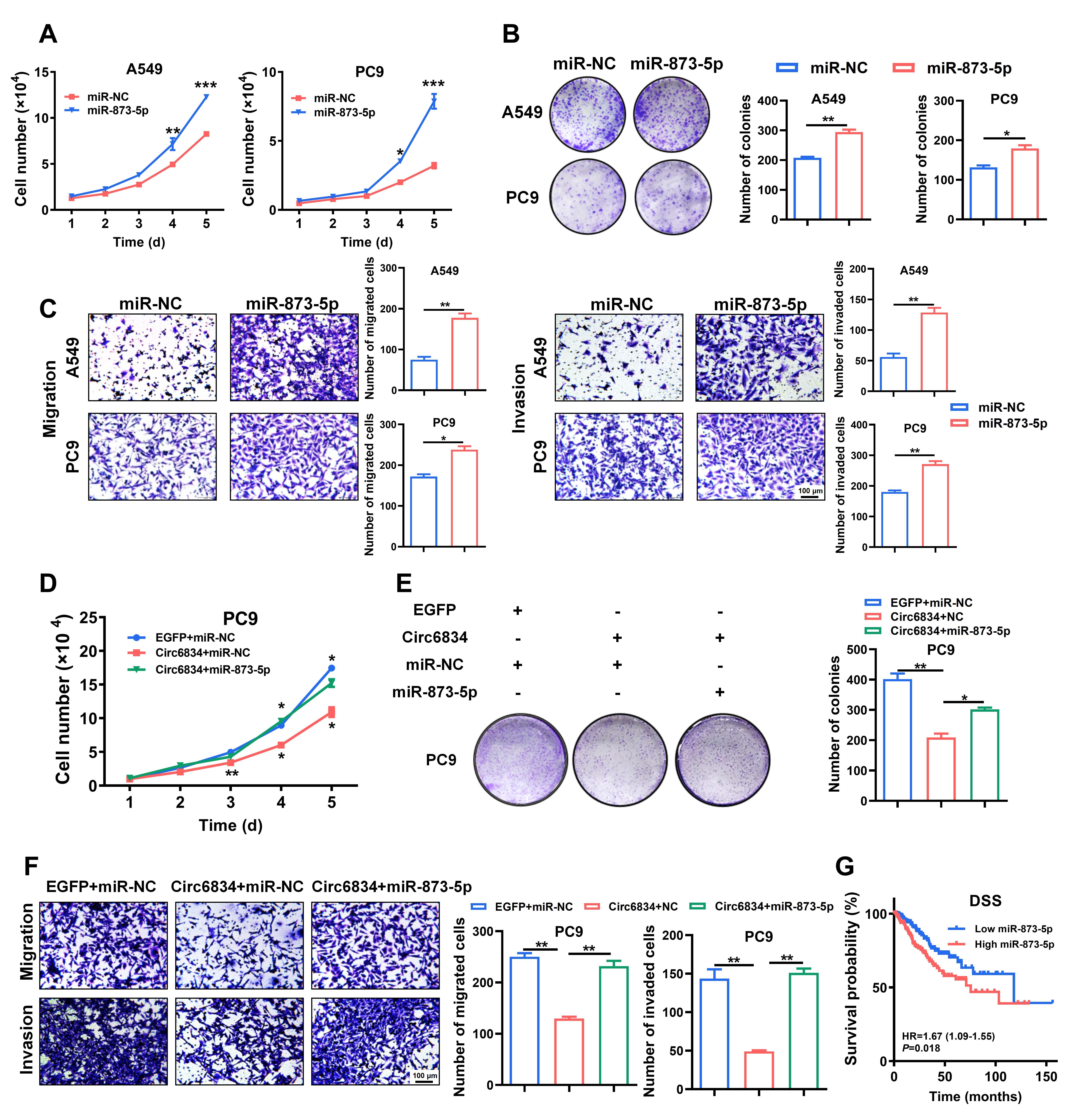


**Supplementary Fig. 8** Circ6834 reverses the cancer-promoting effects of miR-873-5p. **A-C** Cell growth curves (**A**), colony formation assays (**B**), and Transwell migration and Matrigel invasion assays (**C**) for miR-873-5p mimics in NSCLC cells. **D-F** Cell growth curves (**D**), colony formation assays (**E**), and Transwell migration and Matrigel invasion assays (**F**) for circ6834 and miR-873-5p overexpression in PC9 cells. **G** TCGA databsse analysis for disease-specific survival time of lung cancer patients with different miR-873-5p expression.


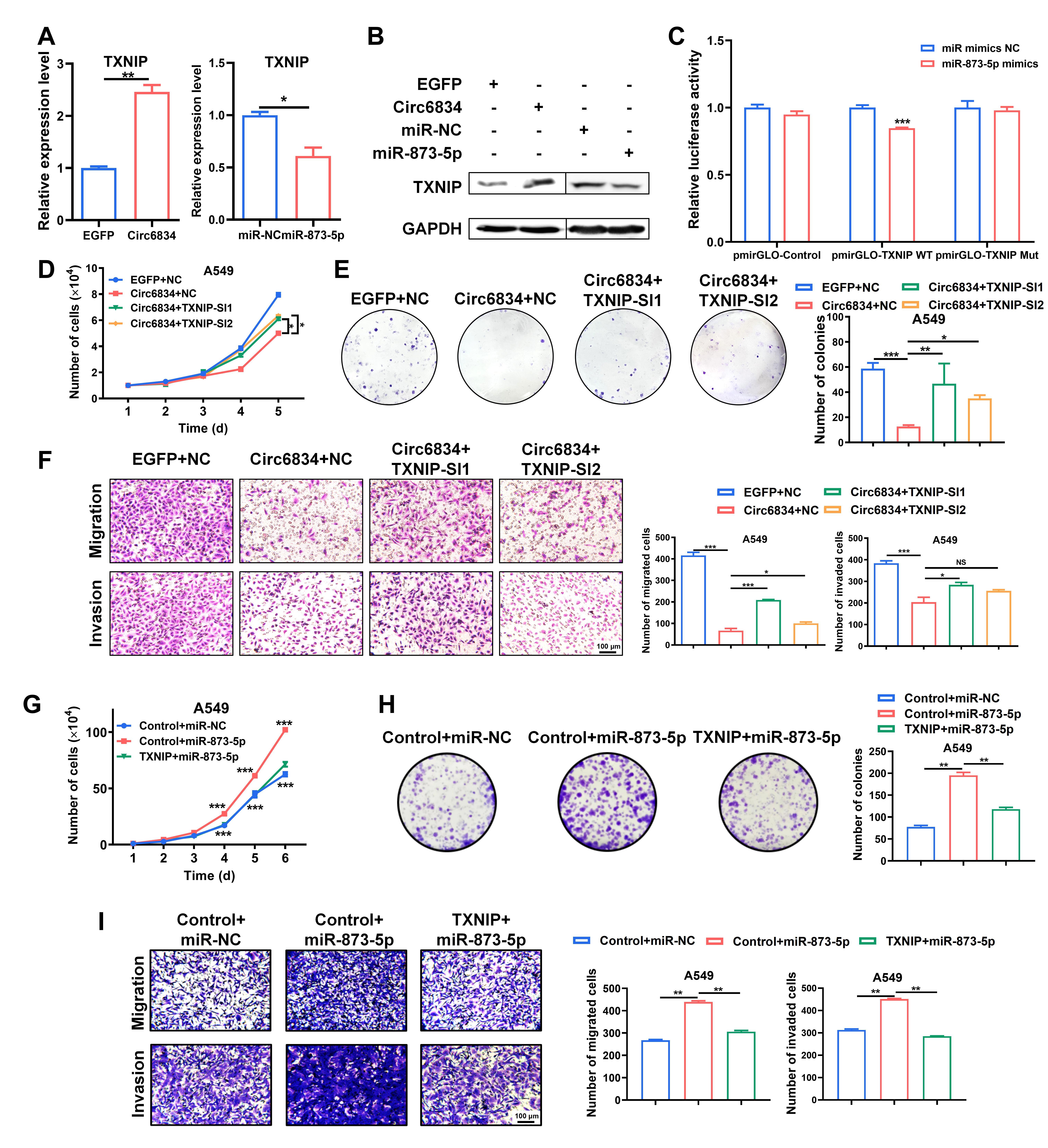


**Supplementary Fig. 9** Circ6834 regulates miR-873-5p/TXNIP axis in NSCLC. **A-B** QRT-PCR (**A**) and western blot (**B**) were performed to detect the expression of TXNIP in PC9 cells with circ6834 or miR-873-5p overexpression. **C** A549 cells were transfected with TXNIP WT or Mut plasmids and miR-873-5p mimics as indicated. Relative luciferase activity was detected and normalized to the control. **D-F** Cell growth curves (**D**), colony formation assays (**E**), and Transwell migration and Matrigel invasion assays (**F**) of circ6834 overexpressing and TXNIP knockdown A549 cells. **G-I** Cell growth curves (**G**), colony formation assays (**H**), and Transwell migration and Matrigel invasion assays (**I**) of A549 cells cotransfected with miR-873-5p mimics and TXNIP plasmid.

**Supplementary tables**

**Supplementary table. 1** The sequences of primers

| Gene | Forward primers | Reverse primers |
| --- | --- | --- |
| β-actin | CACGAAACTACCTTCAACTCC | CATACTCCTGCTTGCTGATC |
| Circ6834 | TGTTGGATTCAGGAGCTTGT | GCTTGGGATGTTTCCATAGT |
| TRIM25 | AAAGCCACCAGCTCACATCCGA | GCGGTGTTGTAGTCCAGGATGA |
| TXNIP | GGTCTTTAACGACCCTGAAAAGG | ACACGAGTAACTTCACACACCT |
| AHNAK | GTGGGTGCCACCATCTACTT | GGTGGAGACTGAAACTGCCC |
| EPB41L5 | TGTACCACCTGGACCTGATTGA | ACCATCCAACCAATGTGCTACT |
| QKI | TAAATCACCAACAGCCCAGG | GTTCCGTTTGGCATGACAGC |

**Supplementary table. 2** The sequences of siRNAs and miRNA mimics

| Gene | Sense | Antisense |
| --- | --- | --- |
| Negative control | UUCUCCGAACGUGUCACGUTT | ACGUGACACGUUCGGAGAATT |
| Si-circ6834-1 | CAUCAUCGGACUUGUAAGATT | UCUUACAAGUCCGAUGAUGTT |
| Si-circ6834-2 | CAUCGGACUUGUAAGACUUTT | AAGUCUUACAAGUCCGAUGTT |
| Si-TXNIP-1 | GGAUCUGGUGGAUGUCAAUTT | AUUGACAUCCACCAGAUCCTT |
| Si-TXNIP-2 | GCCACACUUACCUUGCCAATT | UUGGCAAGGUAAGUGUGGCTT |
| Si-AHNAK | GCCCUGAAUUCAAGAUCAATT | UUGAUCUUGAAUUCAGGGCTT |
| Si-QKI-1 | GGGACCUAUUGUUCAGUUATT | UAACUGAACAAUAGGUCCCTT |
| Si-QKI-2 | GGAGCAUCUAAAUGAAGAUTT | AUCUUCAUUUAGAUGCUCCTT |
| miR-873-5p mimics | GCAGGAACUUGUGAGUCUCCU | AGGAGACUCACAAGUUCCUGC |
